# AtRsmD Is Required for Chloroplast Development and Chloroplast Function in *Arabidopsis thaliana*

**DOI:** 10.3389/fpls.2022.860945

**Published:** 2022-04-25

**Authors:** Zi-Yuan Wang, Wan-Tong Qu, Tong Mei, Nan Zhang, Nai-Ying Yang, Xiao-Feng Xu, Hai-Bo Xiong, Zhong-Nan Yang, Qing-Bo Yu

**Affiliations:** Shanghai Key Laboratory of Plant Molecular Sciences, College of Life Sciences, Shanghai Normal University, Shanghai, China

**Keywords:** AtRsmD, chloroplast, *rRNA* methylation, ribosome, photosynthesis

## Abstract

AtRsmD was recently demonstrated to be a chloroplast *16S rRNA* methyltransferase (MTase) for the m^2^G915 modification in Arabidopsis. Here, its function of AtRsmD for chloroplast development and photosynthesis was further analyzed. The *AtRsmD* gene is highly expressed in green photosynthetic tissues. AtRsmD is associated with the thylakoid in chloroplasts. The *atrsmd-2* mutant exhibited impaired photosynthetic efficiency in emerging leaves under normal growth conditions. A few thylakoid lamellas could be observed in the chloroplast from the *atrsmd-2* mutant, and these thylakoids were loosely organized. Knockout of the *AtRsmD* gene had minor effects on chloroplast ribosome biogenesis and RNA loading on chloroplast ribosomes, but it reduced the amounts of chloroplast-encoded photosynthesis-related proteins in the emerging leaves, for example, D1, D2, CP43, and CP47, which reduced the accumulation of the photosynthetic complex. Nevertheless, knockout of the *AtRsmD* gene did not cause a general reduction in chloroplast-encoded proteins in Arabidopsis grown under normal growth conditions. Additionally, the *atrsmd-2* mutant exhibited more sensitivity to lincomycin, which specifically inhibits the elongation of nascent polypeptide chains. Cold stress exacerbated the effect on chloroplast ribosome biogenesis in the *atrsmd-2* mutant. All these data suggest that the AtRsmD protein plays distinct regulatory roles in chloroplast translation, which is required for chloroplast development and chloroplast function.

## Introduction

In all living organisms, ribosomal RNAs (*rRNAs*) constitute the functional center of the ribosome. These RNAs are extensively modified, and the modified nucleotides are usually located in the regions crucial to the function of the ribosome (Urlaub et al., 1997). In *Escherichia coli*, the majority of modified nucleotides in *rRNAs* belong to various types of base and ribose methylated residues (Sergiev et al., 2011), and there are 11 modified nucleotides in *16S rRNA* (Andersen and Douthwaite, 2006). The three-dimensional locations of these modifications on ribosome crystal structures indicate that these nucleotides are distributed within several discrete regions related to essential ribosomal functions (Brimacombe et al., 1993; Ban et al., 2000; Wimberly et al., 2000; Harms et al., 2001; Yusupov et al., 2001; Schuwirth et al., 2005). *rRNA* modifications play important roles in efficient protein synthesis (Krzyzosiak et al., 1987; Green and Noller, 1999; Khaitovich et al., 1999). The corresponding enzymes for these sites have been determined and characterized in *E. coli* (Andersen and Douthwaite, 2006). For example, the *yebU* gene encodes a putative m^5^C RNA MTase for the methylation of nucleotide 1407 in *16S rRNA*, and the *yebU* knockout strain displays slower growth and reduced fitness in competition with wild-type cells. The *RsmH* gene encodes a conserved MTase that is a specific AdoMet-dependent MTase responsible for the N(4)-methylation of C1402 in *16S rRNA* in almost all species of bacteria (Wei et al., 2012). Two *Escherichia coli* guanine-N_2_ (m2G) MTases, *rsmC* and *rsmD*, modify nucleotides G1207 and G966 in *16S rRNA*, respectively, and they possess a common MTase domain related to the YbiN family of hypothetical MTases in their C-terminus and a variable region in their N-terminus. Knockout of either *rsmC* or *rsmD* results in a mild reduction in cellular growth in *E. coli* (Bujnicki and Rychlewski, 2002; Kimura and Suzuki, 2010). Characterization of these MTase for *16S rRNA* methylation has provided a comprehensive understanding of ribosome biogenesis in prokaryotes.

Chloroplasts in plant cells are derived from an endosymbiotic event in which a photosynthetic cyanobacterium was engulfed by an early eukaryotic cell (Keeling, 2013). During host-endosymbiont coevolution, the chloroplast retained a small-scale genome, while many genes were transferred to the nuclear genome (Abdallah et al., 2000). The chloroplast has both the transcriptional and translational machinery for the expression of chloroplast genes. The translational machinery is a bacterial-type 70S ribosome that is composed of a large 50S subunit and a small 30S subunit. The small 30S subunit comprises 25 ribosomal proteins and chloroplast *16S rRNA*, while the 50S large subunit consists of 33 ribosomal proteins and three other types of chloroplast *rRNAs* (*23S rRNA*, *4.5S rRNA*, and *5S rRNA*) (Graf et al., 2017; Zoschke and Bock, 2018). Not only do chloroplast ribosomal proteins exhibit high similarities to their counterparts in *E. coli*, but *rRNA* constituents are also conserved between chloroplasts and *E. coli* (Zoschke and Bock, 2018). Analogous to methylation in *E. coli rRNAs*, this modification occurs in chloroplast *rRNAs*, and the modification sites and the corresponding enzymes are highly conserved (Zou et al., 2020; Manduzio and Kang, 2021; Ngoc et al., 2021a,b). In contrast, research on *rRNA* modification in chloroplasts lags behind that in *E. coli*. Two independent groups recently reported that the chloroplast-localized CMAL protein, an ortholog of the bacterial MraW/RsmH protein, is involved in the m^4^C methylation of C1352 in chloroplast *16S rRNA*, which plays essential roles in chloroplast development and hormonal responses in higher plants (Zou et al., 2020; Ngoc et al., 2021a). Ngoc et al. (2021b) reported that chloroplast-localized AtRsmD, an ortholog of the MTase for the N_2_-methylguanosine (m^2^G) modification of *16S rRNA* in *E. coli*, is involved in m^2^G methylation at position 915 in chloroplast *16S rRNA* in Arabidopsis. The *atrsmd* mutant exhibited no observable differences in seed germination or seedling growth when grown under normal growth conditions; however, the *atrsmd* mutant was hypersensitive to both cold stress and translation inhibitors (Ngoc et al., 2021b). This finding indicates that AtRsmD is a chloroplast *16S rRNA* MTase for the m^2^G915 modification and that it plays a role in the adaptation of Arabidopsis to cold stress (Ngoc et al., 2021b). Nevertheless, the role of the AtRsmD protein in chloroplast development remains to be further explored.

In this study, we further characterized the *AtRsmD* gene and the corresponding *AtRsmD* deletion mutant, *atrsmd-2*. Knockout of the *AtRsmD* gene impaired chloroplast development and reduced photosynthetic efficiency in emerging leaves in Arabidopsis under normal growth conditions. It did not cause in a general reduction in chloroplast-encoded proteins in Arabidopsis when grown under normal culture conditions but affected the amounts of photosynthetic proteins, such as D1, D2, CP43, and CP47. This reduction interrupted the accumulation of the photosynthetic complex in the *atrsmd-2* mutant. These data suggested that the AtRsmD protein plays distinct regulatory roles in the rapid synthesis of photosynthesis-related proteins, which are required for chloroplast development and chloroplast function.

## Materials and Methods

### Plant Materials and Growth Conditions

The *Arabidopsis thaliana* Col-0 ecotype was used in this study. The T-DNA insertional line (CS832131) was obtained from the Arabidopsis Biological Resource Centre (ABRC; Ohio State University, United States). Plants were grown in a growth chamber with a 16-h light/8-h dark photoperiod at a constant temperature of 22°C. The light intensity was 120 μmol m^–2^ s^–1^. For growth on agar plates, the seeds were surface-sterilized with 75% alcohol and sown on Murashige-Skoog (MS) medium and 0.7% (w/v) phytoagar with or without the corresponding antibiotics.

For the genomic complementation experiment, the 2,594 bp wild-type genomic fragment of the *AtRsmD* (AT3G28460) gene was amplified with the gene-specific primers listed Supplementary Table 1 using high-fidelity KOD plus polymerases (TOYOBO)^1^ and then subcloned into the modified pCAMBIA1300 binary vector (CAMBIA)^2^ with a three-tandem FLAG tag. The construct was introduced into the *atrsmd-2* mutant via *Agrobacterium tumefaciens* GV3101 (Clough and Bent, 1998). Transgenic lines were screened on MS medium with 80 mg/L hygromycin B (Roche).^3^

### Chlorophyll Fluorescence and Chlorophyll Content Measurements

Chlorophyll fluorescence was determined using an Imaging PAM fluorometer (Walz, Germany). Measurement of chlorophyll fluorescence was performed according to Li et al.’s (2019) report. For chlorophyll content determination, equal fresh weights of Arabidopsis leaves were extracted with equal volumes of 80% (v/v) acetone, and the absorbance of the supernatant was measured at 663 and 646 nm. Both chlorophyll a and b contents were calculated using the equation of Porra (2002).

### Transmission Electron Microscopy Observation

Young primary leaves grown under normal conditions or cold treatments were fixed and embedded as described in a previous report (Yu et al., 2013). Thin sections were prepared with an ultramicrotome and then stained with uranyl acetate and lead citrate. The ultrastructure was examined using a Tecnai Spirit G2 BioTWIN transmission electron microscope.

### Subcellular Localization Analysis

The full-length coding sequence of the *AtRsmD* gene was amplified by high-fidelity KOD plus polymerases (TOYOBO)^4^ with the corresponding gene-specific primers and fused in frame with the enhanced green fluorescence protein (eGFP) of the modified pRIN101:eGFP vector (TOYOBO, see text footnote 4) to produce AtRsmD:eGFP. Both the construct and free pRIN101:eGFP vector were transformed into Arabidopsis protoplasts prepared as described in a previous report, respectively (Yoo et al., 2007). The fluorescence signals were imaged by a laser-scanning confocal microscope (Zeiss, Oberkochen, Germany) with excitation and emission wavelengths of 488 and 545 nm, respectively.

Chloroplast fractions were isolated as described in a previous report (Xiong et al., 2020). Three-week-old leaves from the *AtRsmD:eGFP* stable transgenic lines were homogenized in precooled buffer I (0.33 M sorbitol; 0.02 M Tricine/KOH, pH 8.4; 5 mM EGTA, pH 8.35; 5 mM EDTA, pH 8.0; 10 mM NaHCO_3_). After filtration with a double layer of Miracloth, the homogenate was further filtered through 100- and 40-μm sieves, in order, and the filtrate was centrifuged at 2,000 g for 5 min at 4°C to obtain intact chloroplasts. Following centrifugation, the intact chloroplasts were then resuspended in buffer II (0.33 M sorbitol; 5 mM MgCl_2_; 2.5 M EDTA, pH 8.0; 20 mM HEPES/KOH, pH 7.6) and in buffer III (5 mM MgCl_2_-6H_2_O, 25 M EDTA pH 8.0, 20 mM HEPES/KOH pH 7.6), successively. After centrifugation at 4°C, the supernatant contained stroma, while the sediment contained the thylakoid fractions. The corresponding antibodies were used to perform immunoblotting analysis.

### Sequence Alignment and Phylogenetic Tree Construction

Protein sequences were aligned with ClustalW, and the alignment results were displayed with the BoxShade Server.^5^ The phylogenetic tree was constructed and tested using MEGA 3.1^6^ based on the neighbor-joining method.

### Nucleic Acid Isolation, cDNA Synthesis, and qPCR Analysis

For genomic DNA isolation, samples were homogenized in lysis buffer [200 mM Tris-HCl, pH 7.5; 25 mM NaCl; 25 mM EDTA; and 0.5% (w/v) SDS], and the homogenate was isolated with phenol/chloroform (1:1, v/v). After centrifugation, the supernatant was collected, and genomic DNA was precipitated by adding ice-cold isopropyl alcohol. After washing with 70% (v/v) ethanol, the genomic DNA was air-dried and dissolved in double-distilled water. For total RNA isolation, samples were ground in liquid nitrogen, and total RNA was isolated using an RNA isolation kit (Tiangen)^7^ according to the manufacturer’s instructions.

Total RNA (5 μg) was used to synthesize first-strand cDNA with Trans-Script^®^ Fly First-Strand cDNA Synthesis Super-Mix. Quantitative PCR (qPCR) analysis was performed using the gene-specific primers listed in Supplementary Table 1 and SYBRGREEN I master mix reagent (Toyobo, see text footnote 1) on a real-time RT–PCR system (ABI7300, United States). Reactions were performed in triplicate for each sample, and expression levels were normalized against *TUBLIN4*.

### Polysome Association Analysis

Polysome association assays were performed as described previously (Zhang et al., 2018). Total extracts from the young leaves of 2-week-old plants were fractionated in 15–55% sucrose gradients through centrifugation at 45,000 rpm (246,000 g) at 4°C for 65 min. After centrifugation, 10 fractions (0.5 mL each fraction) were collected successively from the sucrose gradients. Then, the total RNA in each fraction was isolated, and an equal proportion of RNA was separated using RNA gel blot analysis.

### RNA Gel-Blot Hybridization

RNA hybridization was performed according to the Roche manual as described previously (Yu et al., 2013). Total RNA (5 μg) from the wild type and *atrsmd-2* mutant was separated using formamide denaturing agarose gel electrophoresis, transferred onto nylon membranes (MILLIPORE), and subjected to RNA gel blotting with digoxigenin (DIG)-labeled nucleic acid probes (DIG Easy Hyb system; Roche). The probes were prepared using a PCR DIG synthesis kit (Roche, see text footnote 3) with the specific primers listed in Supplementary Table 1. Total RNA was detected by ethidium bromide staining, and the signals on the RNA gel blot were visualized with a LuminoGraph WSE-6100 (ATTO).

### Chloroplast Blue Native PAGE and Two-Dimensional Analysis

Chloroplast BN-PAGE and 2-D analysis were performed according to a previous report (Zhang et al., 2018). Briefly, 1 g of 2-week-old seedlings was homogenized in precooled HMSN buffer (0.4 M sucrose, 10 mM NaCl, 5 mM MgCl_2_-6H_2_O, and 10 mM HEPES) and then filtered through a multilayer microcloth. The isolated thylakoid pellets were resuspended in buffer [25 mM Bis-Tris-HCl, pH 7.0, 1% n-dodecyl b-D-maltoside (w/v), and 20% glycerol (w/v)] at 1.0 mg chlorophyll/mL and incubated at 4°C for 5 min followed by centrifugation at 12,000 g for 10 min. After one-tenth volume of loading buffer [100 mM Bis-Tris-HCl, pH 7.0, 0.5 M 6-amino-n-caproic acid, 5% Serva blue G (w/v), and 30% glycerol (w/v)] was added to the supernatant, and the samples were separated on 0.75-mm 4–12% acrylamide gradient gels in a Tannon vertical electrophoresis device at 4°C. For two-dimensional analysis, excised BN-PAGE lanes were soaked in SDS sample buffer for 30 min and layered onto 1-mm 10% SDS polyacrylamide gels containing 6 M urea. After electrophoresis, the proteins were stained with Coomassie bright blue.

### Total Protein Extraction and Immunoblotting Analysis

Total protein was isolated from the young leaves of 2-week-old Arabidopsis seedlings according to a previous report (Zou et al., 2020). Briefly, samples were ground in extraction buffer [25 mM Tris-HCl (pH 7.9), 50 mM NaCl, 1 mM EDTA, 5% glycerol (v/v), and 2% SDS] and subsequently centrifuged at 12,000 g for 10 min. The total protein in the supernatant was quantified using the DC Protein Assay Kit according to the manufacturer’s instructions (Bio–Rad Laboratories). Approximately 10 μg of total protein was separated on 10% SDS polyacrylamide gels and transferred onto PVDF membranes. Immunoblotting analysis was performed with specific primary antibodies, and the signals from the secondary conjugated antibodies were detected with enhanced chemiluminescence and a TANNON imaging system.

## Results

### Knockout of the *AtRsmD* Gene Impaired Photosynthetic Efficiency in Arabidopsis

**FIGURE 1:**
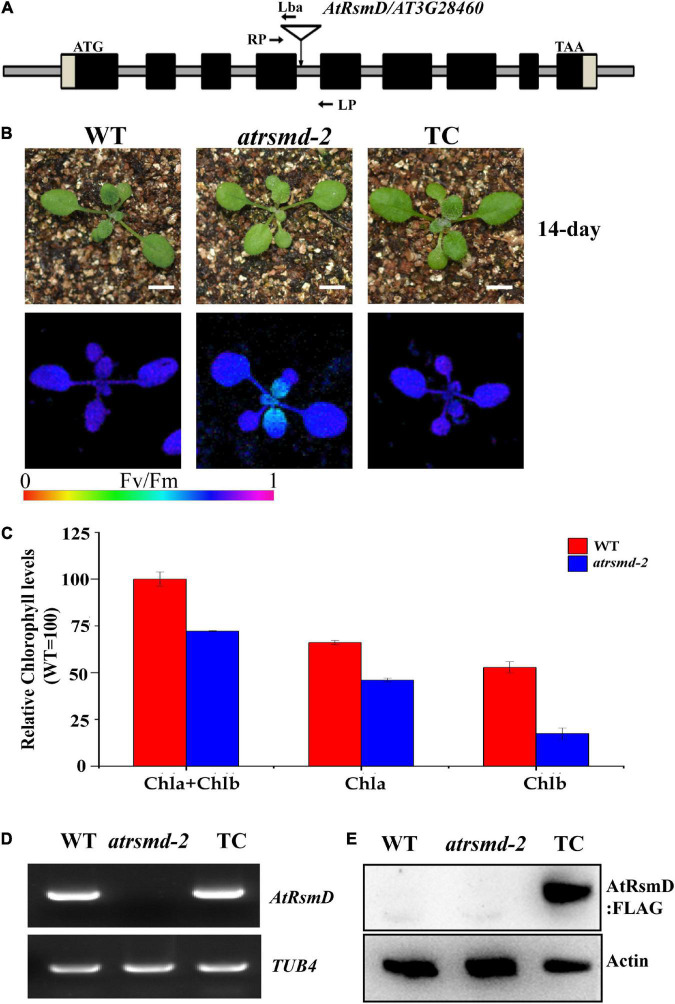
Knockout of the *AtRsmD* gene impaired photosynthetic efficiency in Arabidopsis. **(A)** Schematic illustrating the genomic structures of *AtRsmD* and the location of the T-DNA insertion. Black boxes and striped boxes indicate exons and introns, respectively. The T-DNA insertion site is indicated by an inverted triangle. Lba represents the left border primer of the T-DNA insertion. LP and RP represent the left and right genomic primers around the T-DNA insertion site, respectively. **(B)** Phenotype and *Fv/Fm* values of 14-day-old seedlings the wild type (WT), *atrsmd-2* mutant and complemented lines (TC). Bar represents 1 cm. **(C)** Chlorophyll content in the emerging leaves from the 14-day-old wild type and *atrsmd-2* mutant seedlings. **(D)** RT–PCR analysis of the *AtRsmD* gene in the wild type, *atrsmd-2* mutant, and complemented line (TC). *Tubulin 4* was used as a control. **(E)** Immunoblotting analysis of AtRsmD:FLAG in the wild type, *atrsmd-2* mutant, and complemented line (TC) with the anti-FLAG antibody. Actin was used as a control.

The chlorophyll fluorescence parameter *Fv/Fm* reflects the maximum quantum efficiency of photosystem II (PSII) photochemistry and has been widely used for early stress detection in plants. To obtain novel insights into the regulation of photosynthetic efficiency in Arabidopsis, we screened Arabidopsis T-DNA insertion lines on a large scale to detect their *Fv/Fm* values. We found that the *Fv/Fm* value of the homozygous T-DNA insertion line CS832131 was only 0.643 ± 0.02, which was lower than the 0.788 ± 0.02 value found for the control (Figures 1A,B). The homozygous mutants exhibited no obviously visible phenotype (Figure 1B). Measurement of the total chlorophyll contents indicated that the chlorophyll contents in the homozygous mutant were slightly lower than those in the wild type (Figure 1C). PCR sequencing analysis confirmed that the T-DNA is inserted in the fourth intron of the *AT3G28460* gene. RT-PCR analysis showed that the full-length transcript of the *AT3G28460* gene was not detected in the homozygous mutant but was detected in the wild type (Figure 1D), which demonstrated that the line is another mutant for the locus. Therefore, we named the line as the *atrsmd-2* mutant, while a previously reported line (Ngoc et al., 2021b) was named as the *atrsmd-1* mutant.

To verify that the *AT3G28460* locus is responsible for the impaired photosynthetic efficiency in the *atrsmd-2* mutant, we produced a construct carrying the genomic sequence of the *AT3G28460* gene fused with the coding sequence of a three-tandem FLAG tag before its stop codon that was driven by its native promoter, and we transformed it into the *atrsmd-2* mutant *via Agrobacterium tumefaciens* (Clough and Bent, 1998). Three independent transgenic lines carrying the *AtRsmD* genomic fragment were identified to have the *atrsmd-2/-* genotype. RT-PCR results indicated that *AtRsmD* transcripts could be detected in the complemented line (Figure 1D). Immunoblotting analysis detected a protein with the predicted size of 35 kDa in the complemented line, but this protein was absent in both the wild type and *atrsmd-2* mutant (Figure 1E). The values of *Fv/Fm* in the complemented line were restored to the levels of the wild type (Figure 1B). These data showed that knockout of the *AtRsmD* gene impaired the photosynthesis efficiency of photosystem II under normal conditions. Taken together, our data demonstrated that the AtRsmD:FLAG fusion protein is able to complement the *atrsmd-2* mutant, and the fusion protein is functional.

### Expressional Pattern of the *AtRsmD* Gene and Subcellular Localization of Its Product in Arabidopsis

**FIGURE 2:**
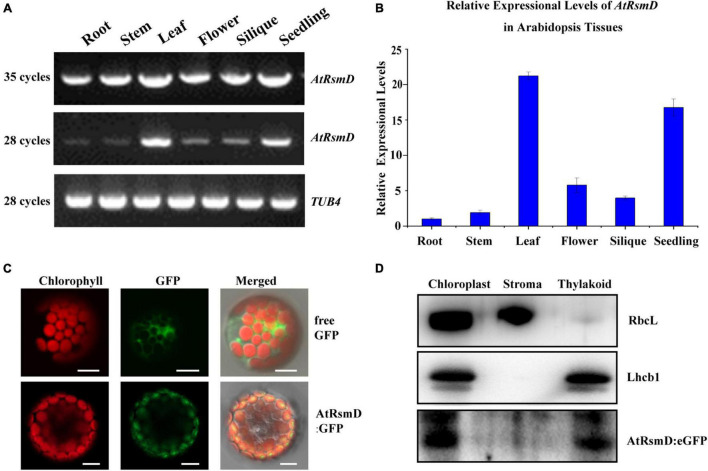
Expression pattern of the *AtRsmD* gene and subcellular localization of its product in Arabidopsis. **(A)** Semi-RT–PCR and **(B)** RT–qPCR analysis of *AtRsmD* mRNA in different Arabidopsis tissues and organs, including roots, stems, leaves, flowers, siliques and seedlings. *Tubulin 4* was used as a control. **(C)** Localization of the AtRsmD:eGFP fusion protein in Arabidopsis protoplasts. Free eGFP in Arabidopsis protoplasts was used as a control. Green fluorescence shows eGFP fluorescence, red fluorescence indicates chloroplast autofluorescence, and orange/yellow fluorescence shows overlapping images of the two types of fluorescence. Scale bars: 5 μm. **(D)** The AtRsmD:eGFP protein is associated with thylakoid membranes in chloroplasts. The fractionation of Arabidopsis seedling chloroplasts was performed with anti-GFP, anti-Lhcb1, and anti-RbcL antibodies.

To examine the expression pattern of the *AtRsmD* gene in different tissues, we performed reverse-transcription (RT)-PCR analysis of various tissues from wild-type plants. Our results showed that the *AtRsmD* transcripts were present in all tissues (Figures 2A,B). The *AtRsmD* transcripts were much more abundant in seedlings and leaves than in the other tissues. These data indicated that the *AtRsmD* gene was highly expressed in these green tissues.

TargetP software predicted that the AtRsmD protein contains a potential chloroplast transpeptide at the N-terminus,^8^ and a very recent study demonstrated that the AtRsmD protein is localized to the chloroplast (Ngoc et al., 2021b). We made a construct in which the full-length encoding sequence of the AtRsmD protein was fused into the coding sequence of eGFP and introduced it into Arabidopsis protoplasts. AtRsmD:eGFP fluorescence colocalized with chlorophyll autofluorescence (Figure 2C), demonstrating that the AtRsmD protein is localized to the chloroplast. We also checked whether AtRsmD is associated with thylakoids. Chloroplast stroma and thylakoids were isolated from the stable AtRsmD:eGFP transgenic lines, and then immunoblotting analysis was carried out with the corresponding antibodies. Our immunoblotting analysis showed that AtRsmD:eGFP was detected in both chloroplasts and thylakoid fragments (Figure 2D). All these data showed that the AtRsmD protein is associated with thylakoids in chloroplasts.

### Absence of the *AtRsmD* Protein Impaired the Accumulation of Chloroplast-Encoded Photosynthetic Proteins

**FIGURE 3:**
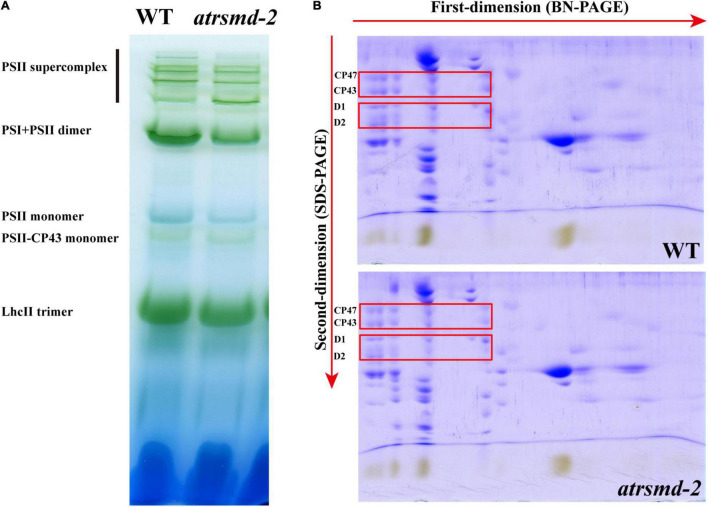
Analysis of photosystem complexes from the wild-type and *atrsmd-2* mutant grown under normal growth conditions. **(A)** BN-gel analysis of the thylakoid membrane protein complexes in the wild type (WT) and the *atrsmd-2* mutant. Representative unstained BN-PAGE gel electrophoresis is shown. Thylakoid membranes from the wild type (WT) and *atrsmd-2* mutant were solubilized with 1% DM and separated by native PAGE. A sample with an equal amount of chlorophyll (16 μg) was loaded in each lane. The bands for supercomplexes are marked on the left. **(B)** 2-D, BN/SDS-urea-PAGE analysis of the thylakoid membrane complexes. Thylakoid complexes were separated by BN-PAGE and further subjected to 2D SDS–PAGE. The gels were stained with Coomassie Brilliant Blue. The locations of the plastid-encoded core proteins of PSII (CP43, CP47, D1, and D2) are indicated by red boxes on the gels, following to a previous report (Li et al., 2019).

The *atrsmd-2* mutant exhibited impaired photosynthetic efficiency (Figure 1B), which could be due to reduced protein levels of photosynthetic complex elements. We investigated whether the absence of *AtRsmD* affects the accumulation of photosynthetic complex elements. Thylakoid membranes were solubilized with 1% dodecyl-β-D-maltopyranoside (DM), and then membrane protein complexes were separated by blue native PAGE (BN-PAGE). Our results showed that the amounts of supercomplex elements, such as the PSII supercomplex, PSI-PSII dimer, PSII monomer, and LhcII trimers, were clearly reduced in the *atrsmd-2* mutant compared with those of the wild type (Figure 3A). The complexes resolved by BN-PAGE were then separated into their subunits in the second dimension by electrophoresis on SDS–PAGE gels and stained with Coomassie brilliant blue R 250. We found that the accumulation of the core subunits of PSII, CP43, CP47, D1, and D2 in the complex was clearly reduced in the *atrsmd-2* mutant (Figure 3B) compared with their counterparts in the wild type. These results showed that assembly of the photosynthetic complex was seriously reduced in the *atrsmd-2* mutant.

**FIGURE 4:**
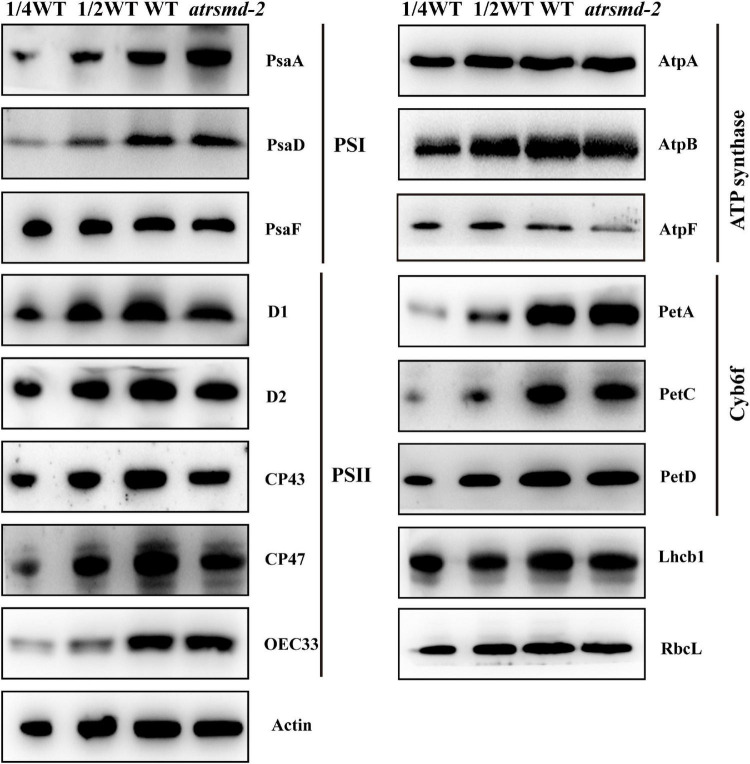
Immunoblot analysis of the photosynthetic proteins from the wild type (WT) and *atrsmd-2* mutant. Total protein samples were prepared from 14-day-old emerging leaves of the wild type and *atrsmd-2* mutant and then separated by SDS–PAGE. Immunoblot analysis of the photosynthetic proteins PsaA, PsaD, PsaF, D1, D2, CP43, CP47, OEC33, AtpA, AtpB, AtpF, PetA, PetC, PetD, Lhcb1, and RbcL using the corresponding antibodies was performed. Total proteins from the WT samples were loaded at three different concentrations (2.5, 5, and 10 μg), and total proteins from the mutant samples were loaded at 10 μg.

To examine the steady-state levels of the thylakoid membrane proteins, immunoblotting analysis was performed with antibodies against specific subunits of the photosynthetic thylakoid membrane complex. Our results showed that the amounts of plastid-encoded photosynthesis-related proteins, including D1, D2, CP43, CP47, and AtpF, were 25–50% those of the wild type (Figure 4). In contrast, other photosynthesis-related proteins, such as AtpA, AtpB, RbcL, PetA, PetD, OEC33, and Lhcb1, accumulated to similar levels as the wild type (Figure 4), while the abundance of the PsaA protein increased in the mutant. These results indicated that knockout of the *AtRsmD* gene interrupted the accumulation of photosynthesis-related proteins, including chloroplast-encoded photosystem II proteins, and affected the assembly of the photosynthetic complex, which probably impaired photosynthetic efficiency in the *atrsmd-2* mutant.

### Absence of *AtRsmD* Affects Chloroplast Transcripts

**FIGURE 5:**
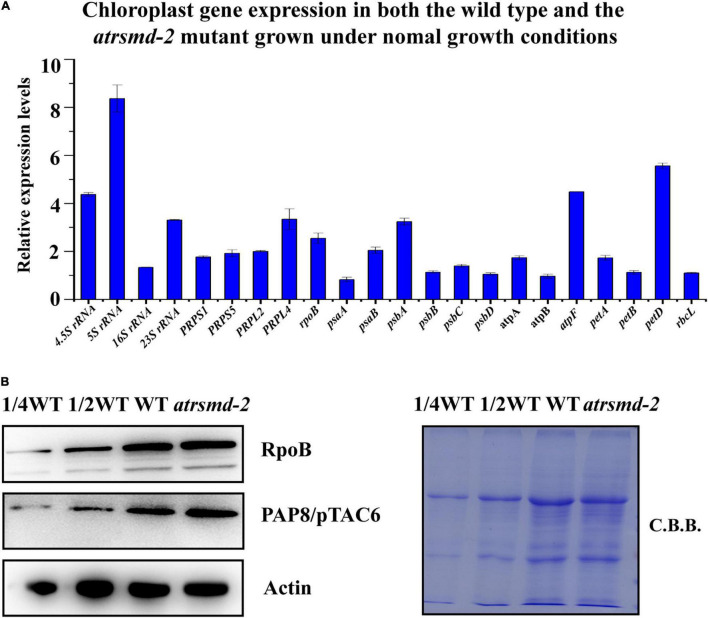
Absence of *AtRsmD* affects chloroplast transcripts in the wild type and *atrsmd-2* mutant. **(A)** Relative expression levels of chloroplast-related genes in the wild type and *atrsmd-2* mutant. **(B)** Immunoblot analysis of the RpoB and PAP8/pTAC6 proteins of the PEP complex from the wild type (WT) and *atrsmd-2* mutant was performed. Total proteins from the WT samples were loaded at three different concentrations (2.5, 5, and 10 μg), and total proteins from the mutant samples were loaded at 10 μg.

Since the amounts of several chloroplast-encoded photosynthetic proteins, including D1, D2, CP43, CP47, and AtpF, were clearly reduced in the *atrsmd* mutant, we investigated whether the absence of the *AtRsmD* gene affected the accumulation of their corresponding chloroplast transcripts through RT–qPCR analysis. Our results showed that the chloroplast transcripts *psbA*, *psbB*, *psbC, psbD*, and *atpF* were 1-6-fold higher in the *atrsmd-2* mutant than those in the wild type (Figure 5A), although the corresponding photosynthetic proteins were clearly reduced in the mutant. Additionally, we observed that the abundances of the chloroplast transcripts *atpA*, *atpB*, *petA*, *petD*, and *rbcL* were increased. We further investigated the accumulation of the PEP complex in the *atrsmd-2* mutant. The amounts of the core subunit, RpoB, and the essential component, PAP8/pTAC6, were checked in this mutant, and our results showed that the abundance of these core subunits in the *atrsmd-2* mutant was slightly increased compared with those in the wild type (Figure 5B). All these results indicated that knockout of the *AtRsmD* gene increased the accumulation of the PEP complex and altered the expression of these chloroplast transcripts in Arabidopsis, which is probably the result of feedback regulation.

### Absence of *AtRsmD* Affects the Accumulation of Chloroplast Ribosomes

**FIGURE 6:**
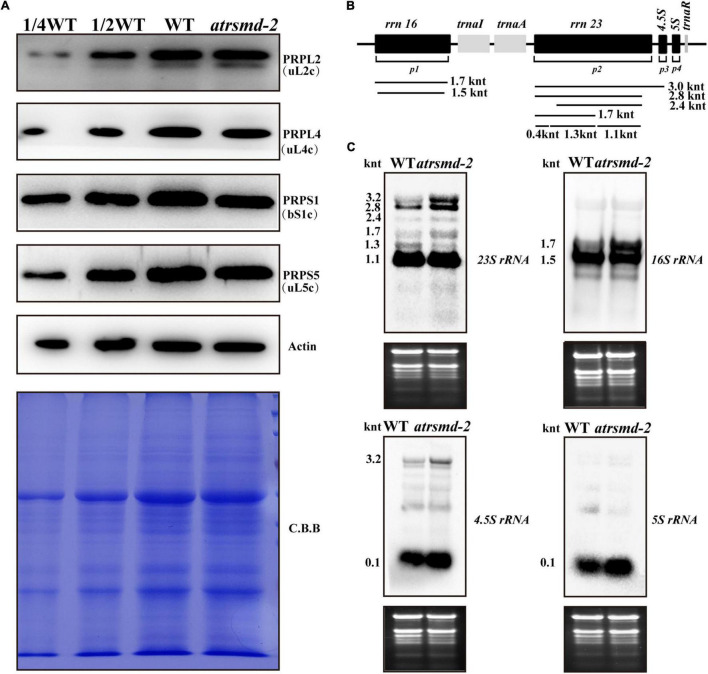
Accumulation of chloroplast ribosomal proteins and *rRNAs* in the wild type and *atrsmd-2* mutant grown under normal conditions. **(A)** Immunoblot analysis of the chloroplast ribosomal proteins from the wild type (WT) and *atrsmd-2* mutant was performed. PRPS1/bS1c,PRPS5/uS5c, PRPL4/uL4c, and PRPL2/uL2cwere checked in the wild type and *atrsmd-2* mutant. Total proteins from the WT samples were loaded at three different concentrations (2.5, 5, and 10 μg), and total proteins from the mutant samples were loaded at 10 μg. **(B)** Schematic diagram of Arabidopsis chloroplast *rRNA* operon and the probe positions used RNA gel blot analyses. Sizes of different transcript forms of chloroplast rRNA are indicated below the diagram. p1, p2, p3, and p4 indicate positions of the probes for *16S rRNA*, *23S rRNA*, *4.5S rRNA*, and *5S rRNA*, respectively. **(C)** RNA gel analysis of chloroplast *rRNA*s in the wild type and *atrsmd-2* mutant. Five micrograms of total RNA was loaded in each lane with the corresponding probes as shown above. Total RNA was stained with ethidium bromide (EtBr) and used as a loading control. The sizes of different transcript forms of chloroplast *rRNAs* are shown.

A previous report indicated that the AtRsmD protein is responsible for the methylation of *16S rRNA* m^2^G915, and this methylation pattern was missing in the knockout line of the *AtRsmD* gene (Ngoc et al., 2021b). We further investigated the possible impacts of the absence of the AtRsmD protein on chloroplast ribosomal biogenesis. The levels of two components of the ribosomal 30S small subunit, PRPS1 (SMALL RIBOSOMAL SUBUNIT 1, bS1c) and PRPS5 (SMALL RIBOSOMAL SUBUNIT 5, uS5c), and two components of the ribosomal 50S large subunit, PRPL4 (LARGE RIBOSOMAL SUBUNIT 4, uL4c) and PRPL2 (LARGE RIBOSOMAL SUBUNIT 2, uL2c), were slightly reduced in the *atrsmd-2* mutant grown under normal growth conditions, compared with those in the wild type (Figure 6A). Then, the levels of four chloroplast *rRNA* transcripts (*16S rRNA*, *23S rRNA*, *5S rRNA*, and *4.5S rRNA*) were also investigated in the *atrsmd-2* mutant using RNA blot analysis (Figures 6B,C). Our results indicated that the amount of the mature form of chloroplast *16S rRNA* (1.5-knt molecule) in the *atrsmd-2* mutant was reduced, whereas the amount of the precursor rRNA (1.7-kb) molecule was increased (Figure 6C). Levels of the 3. 2-, 2. 8-, 2.4- and 1.7-knt *23S rRNA* species were increased in the *atrsmd-2* mutant. Additionally, the level of one shorter form of the 1.3-knt species was decreased (Figure 6C), while the 1.1-knt form accumulated to a level similar as that in the wild type. In contrast, the levels of *4.5S* and *5S rRNA* transcripts were increased in the *atrsmd-2* mutant. These data suggest that knockout of the *AtRsmD* gene affected the accumulation of chloroplast *rRNAs* and chloroplast ribosomal proteins. Nevertheless, knockout of the *AtRsmD* gene has a minor effect on chloroplast ribosome biogenesis.

**FIGURE 7:**
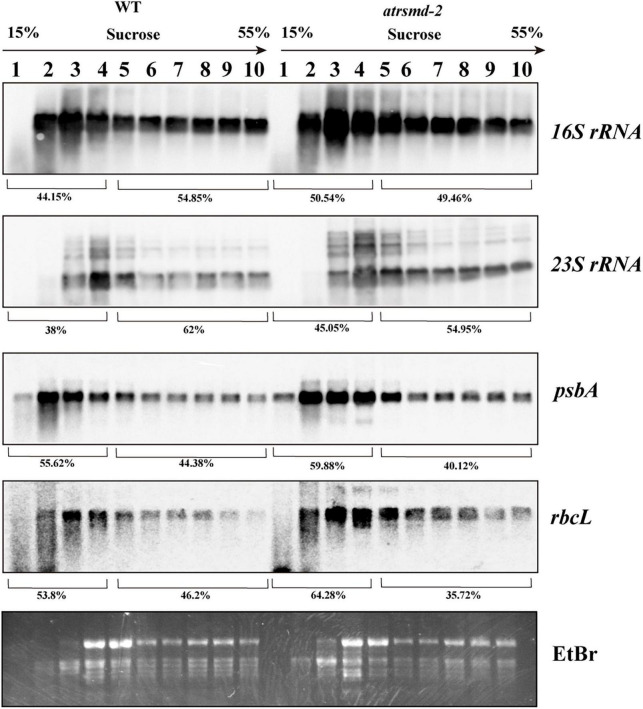
Chloroplast polysome analysis in the wild type and *atrsmd-2* mutant. RNA gel-blot hybridization of fractions obtained following sucrose density-gradient centrifugation under polysome-preserving conditions. Free ribosomes (monosomes) are found in fractions 1–4, whereas fractions 5–10 contain RNA-polysome complexes. Membranes were hybridized with probes specific for *16S rRNA*, *23S rRNA*, *psbA*, and *rbcL*. Ethidium bromide staining was used as a loading control. Signals of the polysomal fractions (fractions 1–4) and monosomal/free RNA fraction (fractions 5–10) were quantified with the ImageJ software, and their relative quantities were indicated as percentage of the total signal over the 10 fractions.

We subsequently investigated the effect of *AtRsmD* gene knockout on chloroplast translation initiation and termination. We examined the association of several plastid-encoded RNAs with chloroplast ribosomes. Polysome-enriched samples were isolated from the emerging young leaves of both the wild-type and *atrsmd-2* mutant under polysome-preserving conditions and further fractionated through sucrose density-gradient centrifugation (Figure 7). Total RNA was isolated from 10 fractions and subjected to denaturing gel electrophoresis and RNA gel blot analyses using selected plastid transcript probes. As shown in Figure 7, we found that the amount of *16S rRNA* in the polysomal fragments was slightly greater than that in the monosomal fragments in the *atrsmd-2* mutant compared with that in the wild type. Similar trends were also observed for other transcripts, including *23S rRNA*, *psbA*, and *rbcL*. These results suggested that the knockout of *AtRsmD* had minor effects on the RNA loading of chloroplast ribosomes.

### Absence of the *AtRsmD* Protein Enhances the Sensitivity to Cold Stress and Chloroplast Translation Inhibitors

A recent study revealed that loss-of-function mutation of the *AtRsmD* gene enhanced its sensitivity to cold stress and prokaryotic translation inhibitors (Ngoc et al., 2021b). Lincomycin (LIN) and chloramphenicol (CAP) specifically inhibit the elongation of nascent polypeptide chains or block mRNA binding to the ribosome in prokaryotes. Here, we also investigated the effect of these two prokaryotic translation inhibitors on the *atrsmd-2* mutant. The *atrsmd-2* mutant was grown on MS medium plates in the presence of different LIN or CAP concentrations under sterile conditions, while the wild-type line was used as a control. Five days after germination, the *atrsmd-2* mutant clearly exhibited hypersensitivity to lincomycin, as indicated by the enhanced loss of leaf coloration relative to the wild-type control. In contrast, the *atrsmd-2* mutant is less sensitive to chloramphenicol than it is to lincomycin. Quantification of chlorophyll levels showed that LIN-treated *atrsmd-2* seedlings accumulated significantly less of these pigments than wild-type seedlings (Supplementary Figure 1). These results demonstrated that knockout of *AtRsmD* enhanced the sensitivity to chloroplast translation inhibitors, especially lincomycin, indicating that the *AtRsmD* knockout line has compromised chloroplast ribosome activity.

**FIGURE 8:**
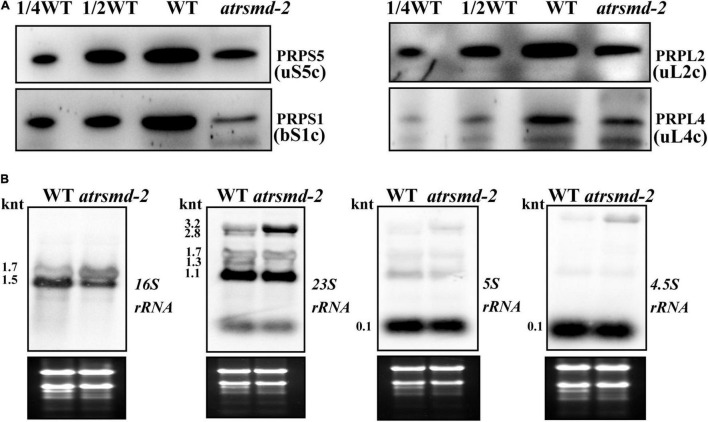
Accumulation of chloroplast ribosomal proteins and chloroplast *rRNAs* in the wild type and *atrsmd-2* mutant treated with cold stress. **(A)** Immunoblot analysis of chloroplast ribosomal proteins, including PRPS1/bS1c, PRPS5/uS5c, PRPL4/uL4c and PRPL2/uL2c, from the wild type (WT) and *atrsmd-2* mutant treated with cold stress. Total proteins from the WT samples were loaded at three different concentrations (2.5, 5, and 10 μg), and total proteins from the mutant samples were loaded at 10 μg. **(B)** RNA gel analysis of chloroplast *rRNA* species in the wild type and *atrsmd-2* mutant treated with cold stress. Total RNA (5 μg) was loaded in each lane with the corresponding probes as shown above.

We also investigated whether the *atrsmd-2* mutant is sensitive to cold stress. Two-week-old *atrsmd-2* mutants grown at 22°C were transferred to 4°C and grown for another 2 weeks, while the wild-type line was used as a control. For the *atrsmd-2* mutant, yellowing became visible after 1 week of cold treatment and only occurred in the emerging leaves (Supplementary Figure 2A). In contrast, the wild type did not show obvious yellowing of the leaves (Supplementary Figure 2A). Transmission electron microscopy observations showed that a few grana and thylakoids could be observed in the chloroplasts of the *atrsmd-2* mutant, and these membrane systems did not connect well. In contrast, the well-organized stroma and thylakoid systems could be observed in the wild-type chloroplasts (Supplementary Figure 2B). Chloroplasts from pale green leaves have more scattered grana structures in the *atrsmd-2* mutant, and the thylakoid membranes were loosely organized. In contrast, well-organized thylakoids could be observed in the wild type (Supplementary Figure 2B). We then checked the abundance of photosynthesis-related proteins, and our results revealed that the amounts of the photosynthesis-related proteins PetD, D1, CP43, OEC33, and AtpF were reduced to 10–50% of those in the wild-type (Supplementary Figure 2C). These results showed that the accumulation of photosynthesis-related proteins was reduced in the *atrsmd-2* mutant under cold stress conditions. We further examined the amount of chloroplast ribosome proteins, including PRPS1 (bS1c), PRPS5 (uS5c), PRPL2 (uL2c), and PRPL4 (uL4c), in the wild type and *atrsmd-2* mutant. Our immunoblotting data revealed that the amounts of these chloroplast ribosomal proteins in the *atrsmd-2* mutant were approximately 10–20% of those in the wild type under cold stress (Figure 8A). The levels of chloroplast *rRNA* transcripts were also investigated using RNA blot analysis in the *atrsmd-2* mutant. Our results indicated that the amount of the mature form of chloroplast *16S rRNA* (1.5-knt molecule) in the *atrsmd-2* mutant was half of that in the wild type, whereas the amount of the precursor *rRNA* (1.7-knt) molecule was increased (Figure 8B). The levels of the three 3.2-knt *23S rRNA* species were increased in *atrsmd-2*, while the level of a shorter form of the 1.3-knt transcript was decreased (Figure 8B). The levels of both the *4.5S* and *5S rRNA* transcripts were clearly reduced in the *atrsmd-2* mutant. These data suggest that the effects of *AtRsmD* gene knockout on chloroplast ribosome assembly and chloroplast protein accumulation were exacerbated, which enhances sensitivity to cold stress.

## Discussion

*AtRsmD* encodes a chloroplast-localized MTase for N_2_-methylguanosine (m2G) modification of *16S rRNA* at position 915 in Arabidopsis chloroplasts, which plays a role in the adaptation of Arabidopsis to cold stress. There were no obvious differences in seedling growth in the knockout mutant when grown under normal growth conditions (Ngoc et al., 2021b). This work extends the previous study and further characterizes the *atrsmd-2* mutant. Our current study sheds light on the important roles of the AtRsmD protein in chloroplast development and chloroplast function. Fewer thylakoid membranes were observed in the *atrsmd-2* mutant (Supplementary Figure 2B). Notably, the loss-of-function *atrsmd-2* mutant exhibited impaired photosynthetic efficiency under normal growth conditions, although no visible phenotype could be observed in the *atrsmd-2* mutant (Figure 1). Impaired photosynthesis is a possible consequence of the reduced levels of chloroplast-encoded photosynthesis-related proteins that hindered the assembly of the photosynthetic super complex (Figures 4, 5). Deletion of the *AtRsmD* gene had minor effects on chloroplast ribosome biogenesis and the RNA loading of chloroplast ribosomes (Figures 6, 7), but the effect on chloroplast ribosome biogenesis and chloroplast development was aggravated when the mutated line was treated with cold stress (Figure 8 and Supplementary Figure 2). Our data and those of a recent report (Ngoc et al., 2021b) together suggested that the AtRsmD protein for the m^2^G915 modification of *16S rRNA* plays important regulatory roles in chloroplast development and chloroplast function.

*16S rRNA* is a constituent of the 30S small subunit in the prokaryotic-type 70S ribosome that is widely present in prokaryotes and eukaryotic organelles (mitochondria and chloroplasts). The nucleotide residue of *16S rRNA* tends to be guanosine in bacteria and chloroplasts (Lesnyak et al., 2007), while it is usually uridine in archaea and adenosine in mitochondria (Lesnyak et al., 2007). Accordingly, homologous proteins of *E. coli* RsmD are present in higher plant chloroplasts (Supplementary Figures 3, 4) but are completely absent in both Archaea and Eukarya. The AtRsmD protein shares highly conserved motifs, such as Dx(F/G/Y)xGxG and (D/N/T)PP(F/Y), with those in *E. coli*; these motifs are required for S-adenosyl-L-methionine (SAM) binding (Supplementary Figure 3; Rana et al., 2013; Ngoc et al., 2021b). Importantly, a recent report demonstrated that AtRsmD is a MTase responsible for chloroplast *16S rRNA* m^2^G 915 methylation, which is absent in the *atrsmd-1* mutant (Ngoc et al., 2021b). Although we did not investigate modifications to the *atrsmd-2* mutant in this study, genetic analysis demonstrated that the phenotype of *atrsmd-2* is caused by the deletion of AtRsmD. Thus, it is possible that chloroplast *16S rRNA* m^2^G 915 methylation is lacking in the *atrsmd-2* mutant. Chloroplast ribosomes are attached to the thylakoid membrane (Yamamoto et al., 1981). A chloroplast ribosome proteome study revealed that the homologous protein of RsmD in *Chlamydomonas reinhardtii* comigrated with the 30S ribosome (Westrich et al., 2021). In *E. coli*, the RsmD enzyme methylates the nucleotide of 16S rRNA and is involved the assembly 30S subunit of ribosome (Lesnyak et al., 2007). AtRsmD also methylates 16S *rRNA* in chloroplast ribosomes (Ngoc et al., 2021b). An early plastid nucleoid proteomics study has shown that the homolog protein of RsmD in maize is a plastid nucleoid protein (Majeran et al., 2012). Our result also indicates that AtRsmD is associated with the thylakoids (Figure 2D). The thylakoid-associated localization of AtRsmD protein and the high levels of the *AtRsmD* transcript in green tissues are consistent with its putative function.

**FIGURE 9:**
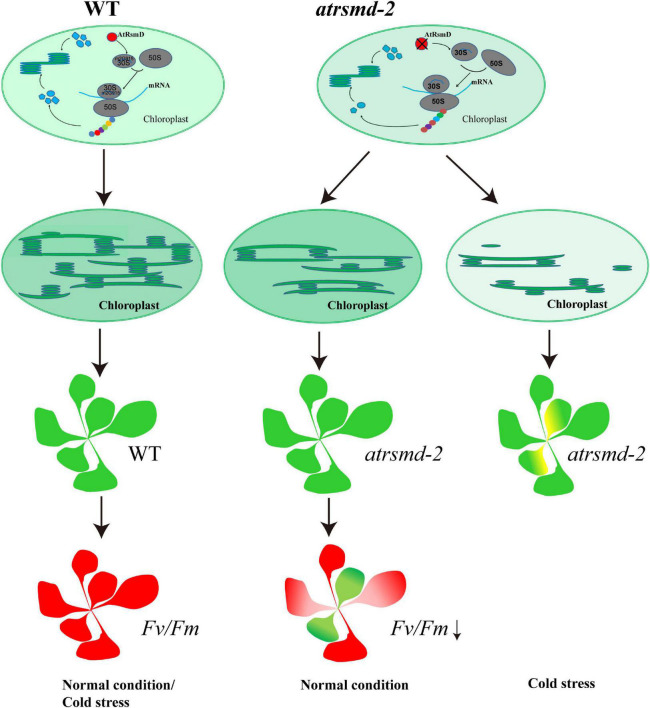
A putative working model for AtRsmD in chloroplasts. AtRsmD is essential for the methylation of the *16S rRNA* m^2^G915 site (Ngoc et al., 2021b), which ensures the rapid synthesis of chloroplast-encoded photosynthetic proteins, such as D1, D2, CP43, and CP47. In the *atrsmd-2* mutant, absence of AtRsmD might reduce the synthesis of these photosynthetic proteins and affect the formation of the thylakoids, which may cause decreased photosynthetic efficiency. Cold stress aggravated the effect of AtRsmD knockout on chloroplast translation, which blocked chloroplast development in the *atrsmd-2* mutant.

Methylation of *rRNAs* has been shown to be a novel cellular regulatory mechanism that is intimately associated with chloroplast translation (Manduzio and Kang, 2021). Multiple nucleotide sites of *16S rRNA* are methylated in *E. coli*, and these methylation sites play important regulatory roles in the function of ribosomes (Carrión et al., 1999; Lesnyak et al., 2007). In *E. coli*, C1352 methylation is essential for viability, and the gene *mraW* encoding the corresponding MTase is essential (Carrión et al., 1999). Additionally, the lack of C1352 methylation in *16S rRNA* (Zou et al., 2020; Ngoc et al., 2021a) clearly reduced chloroplast translation (Zou et al., 2020), which seriously affected plant growth and development. Thus, methylation of the C1352 nucleotide is essential for maintaining chloroplast function. The G966 nucleotide of *E. coli 16S rRNA* is in direct contact with P-site-bound tRNA and forms a direct contact with the tip of the anticodon loop (Lesnyak et al., 2007). The nucleotide sequence of *16S rRNA* is conserved between *E. coli* and chloroplasts (Supplementary Figure 5). Knockout of the *rsmD* gene is viable, and no visible phenotype can be observed in *E. coli* (Jemiolo et al., 1991; Lesnyak et al., 2007; Burakovsky et al., 2012). It seems that no vital function of the ribosome is affected when there is an absence of methylated G966 in *E. coli* (Lesnyak et al., 2007). Similarly, neither germination nor seedling growth of the Arabidopsis *rsmD* mutant were affected under normal growth conditions (Ngoc et al., 2021b). However, our data presented here clearly indicated that the photosynthesis efficiency was reduced in the *atrsmd-2* mutant grown under normal conditions (Figure 1B), although no visible phenotype was observed (Figure 1B). Further observation showed that chloroplast development was impaired in the *atrsmd-2* mutant (Supplementary Figure 2). In accordance with this finding, the chloroplast-encoded photosynthesis-related proteins D1, D2, CP43, CP47, and AtpF were obviously reduced in the *atrsmd-2* mutant, and assembly of the photosynthesis complex was also decreased (Figures 3, 4). Thus, the AtRsmD protein is required for chloroplast development and chloroplast function. In contrast to the effect of the deletion of the CAML protein on chloroplast ribosome biogenesis (Zou et al., 2020), our data indicated that the knockout of the AtRsmD protein had minor effects on chloroplast ribosome biogenesis (Figure 6) and chloroplast RNA loading in Arabidopsis under normal growth conditions (Figure 7). Functional activity of the chloroplast ribosome is retained in the absence of the *16S rRNA* m^2^G915 modification. Lack of the *16S rRNA* m^2^G915 site did not have a major effect on the translation of photosynthetic proteins because the amounts of the chloroplast-encoded photosynthetic proteins D1 and D2 were reduced in the *atrsmd-2* mutant; however, PsaA and PetA were not reduced (Figure 4). This result is different from the observation of the *cmal* mutant (Zou et al., 2020; Ngoc et al., 2021a), in which the absence of C1352 methylation seriously generally affected chloroplast translation events. Noticeably, the nucleotide modification of the G966 nucleotide influences Watson-Crick positions within the modified base in *E. coli*, since the nucleotide is located in the anticodon region (Moazed and Noller, 1990; Lesnyak et al., 2007). Lack of modification interrupts the hydrophobic interaction with the anticodon of the P-site-bound tRNA, which probably affects the fidelity of chloroplast translation. Nevertheless, it is difficult to check the fidelity of chloroplast translation in the *atrsmd-2* mutant at present. Lack of the m^2^G915 modification in the *16S rRNA* might affect the elongation of polypeptide chains because the *atrsmd-2* mutant exhibited more sensitivity to lincomycin, which specifically inhibits the elongation of nascent polypeptide chains (Supplementary Figure 4). It is likely that the m^2^G915 modification of the *16S rRNA* represents a novel regulatory mechanism for translation in chloroplast-encoded photosynthesis proteins rather than a general regulation of chloroplast translation. Mutants in which chloroplast translation is compromised exhibit sensitivity to cold stress (Tokuhisa et al., 1998; Rogalski et al., 2008; Liu et al., 2010; Fleischmann et al., 2011; Zhang et al., 2016; Pulido et al., 2018). Similar to the *atrsmd-1* mutant described in Ngoc et al. (2021b), the *atrsmd-2* mutant was found to be more sensitive to translational inhibitors (Supplementary Figure 1) and cold stress (Supplementary Figure 2) than the wild-type. Further characterization of the *atrsmd-2* mutant treated with cold stress revealed that defects in chloroplast ribosomal biogenesis were aggravated under cold stress (Figure 8). Our study, together with the report by Ngoc et al. (2021b), showed that knockout of the AtRsmD protein for the methylation of 16S rRNA m^2^G915 in chloroplasts reduced the accumulation of several important chloroplast-encoded photosynthetic proteins, which could not meet the needs of rapid chloroplast development and thylakoid membrane formation in emerging leaves, and cold stress exacerbated the effect on chloroplast development (Figure 9). Overall, this study combined with a previous report (Ngoc et al., 2021b) showed that the AtRsmD protein involved in the methylation of *16S rRNA* m^2^G915 in chloroplasts is required for chloroplast development and chloroplast function. This work extends our understanding of the significance of the methylation of chloroplast rRNAs in chloroplast development and chloroplast function.

## Data Availability Statement

The original contributions presented in the study are included in the article/Supplementary Material, further inquiries can be directed to the corresponding author/s.

## Author Contributions

Q-BY designed the research and wrote the manuscript. Z-YW, W-TQ, TM, NZ, and N-YY performed the experiments. Z-NY, H-BX, X-FX, and Q-BY analyzed the data. All authors contributed to the article and approved the submitted version.

## Conflict of Interest

The authors declare that the research was conducted in the absence of any commercial or financial relationships that could be construed as a potential conflict of interest.

## Publisher’s Note

All claims expressed in this article are solely those of the authors and do not necessarily represent those of their affiliated organizations, or those of the publisher, the editors and the reviewers. Any product that may be evaluated in this article, or claim that may be made by its manufacturer, is not guaranteed or endorsed by the publisher.

## Footnotes

1
http://www.toyobo-global.com/

2
http://www.cambia.org

3
http://www.roche.com

4
http://www.toyobo.co.jp

5
http://arete.ibb.waw.pl/PL/html/boxshade.html

6
http://www.megasoftware.net

7
https://www.tiangen.com/

8
www.cbs.dtu.dk/services/TargetP-1.1/index.php

## Supplementary Material

Supplementary Figure 1 Absence of AtRsmD enhances the effects of the translation inhibitors LIN and CAP. **(A)** Representative images of 5-day-old wild-type (WT) and *atrsmd-2* mutant plants germinated on Murashige and Skoog (MS) medium containing the indicated concentrations of LIN. The quantification of the total chlorophyll levels (see section “Materials and Methods”) demonstrates the differences between wild-type and mutant plants grown in the presence of LIN. Relative data are shown (wild-type plants grown in the absence of LIN = 100%), and means ± se values (*n* = 3) are provided. **(B)** Representative images of 5-day-old wild-type (WT) and *atrsmd-2* mutant plants germinated on Murashige and Skoog (MS) medium containing the indicated concentrations of CAP. The quantification of the total chlorophyll levels (see section “Materials and Methods”) demonstrates the differences between wild-type and mutant plants grown in the presence of CAP. Relative data are shown (wild-type plants grown in the absence of CAP = 100%), and means ± se values (*n* = 3) are provided.

Supplementary Figure 2 Chloroplast development in the wild type and *atrsmd-2* mutant treated with cold stress. **(A)** Phenotype of the wild type and *atrsmd-2* mutant treated with cold stress. Bar: 1 cm. **(B)** Chloroplast ultrastructure observation in the wild type and *atrsmd-2* mutant. Bar: 1 μm. **(C)** Immunoblot analysis of the photosynthetic proteins from the wild type (WT) and *atrsmd-2* mutant that were treated with cold stress. Immunoblot analysis of the photosynthetic proteins PsaA, PsaF, D1, CP43, OEC33, AtpA, AtpC, AtpF, PetC, PetD, and RbcL was performed using the corresponding antibodies. Total proteins from the WT samples were loaded at three different concentrations (2.5, 5, and 10 μg), and total proteins from the mutant samples were loaded at 10 μg.

Supplementary Figure 3 Alignment of *E. coli* RsmD homologous proteins in land plants. Alignment of the amino acid sequence of RsmD homologs in land plants. Sequence identifiers for RsmD homologs are as follows: *Arabidopsis thaliana* (NP_189487.2), *Oryza sativa* japonica group (EEE59885.1), and *Escherichia coli* (EGS2103359.1).

Supplementary Figure 4 Phylogenetic analysis of the AtRsmD protein and its orthologs from different species. The orthologous proteins are listed as follows: *Camelina sativa* (XP_010514550.1), *Brassica napus* (XP_013711825.1), *Capsella rubella* (XP_023639147.1), *Arabidopsis lyrata* subsp. *Lyrata* (XP_002877109.1), *Arabidopsis thaliana* (NP_189487.2), *Ricinus communis* (XP_015573725.1), *Vitis vinifera* (XP_002281501.1), *Oryza sativa Japonica Group* (EEE59885.1), *Zea mays* (ACG38583.1), *Physcomitrella patens* (XP_024381071.1), *Selaginella moellendorffii* (EFJ21750.1), *Ostreococcus lucimarinus* CCE9901(XP_001417522.1), *Micromonas pusilla* CCMP1545(XP_003060663.1), *Chloropicon primus* (QDZ19162.1), *Gonium pectoral* (KXZ51239.1), *Chlamydomonas reinhardtii* (XP_042917255.1), *Cyanidioschyzon merolae strain 10D* (XP_005537355.1), *Galdieria sulphuraria* (XP_005703506.1), *Gracilariopsis chorda* (PXF40802.1), *Fischerella thermalis* (WP_102206177.1), *Escherichia coli* (EGS2103359.1), *Oscillatoriales cyanobacterium* MTP1(TAD77760.1), *Synechococcus* sp. *PCC 73109* (WP_062431539.1), *Cyanobacteria* (WP_044151411.1), *Synechocystis* sp. *PCC 7509* (WP_009631812.1), *Nostoc* sp. *106C* (WP_086756145.1), and *Phormidium ambiguum* (WP_073593883.1).

Supplementary Figure 5 Alignment of *16S rRNAs* from different organisms. Sequence identifiers for RsmD homologs are as follows: *Oryza sativa* (MK348618.1), *Brachypodium distachyon* (LR537486.1), *Hordeum vulgare*, (MN171392.1), *Sorghum bicolor* (MK348612.1), *Zea mays* (MK348606.1), *Arabidopsis thaliana* (MK353213.1), *Cyanobacterium* (LN833508.1), *Populus trichocarpa* (AC208048.1), *Glycine max* (DQ317523.1), *Chlamydomonas reinhardtii* (MF083689.2), and *Escherichia coli* (CP056921.1).

## References

[B1] Abdallah F. Salamini F. Leister D. (2000). A prediction of the size and evolutionary origin of the proteome of chloroplasts of *Arabidopsis*. *Trends Plant Sci.* 5 141–142. 10.1016/s1360-1385(00)01574-0 10928822

[B2] Andersen N. M. Douthwaite S. (2006). YebU is a m5C methyltransferase specific for 16 S rRNA nucleotide 1407. *J. Mol. Biol.* 359 777–786. 10.1016/j.jmb.2006.04.007 16678201

[B3] Ban N. Nissen P. Hansen J. Moore P. B. Steitz T. A. (2000). The complete atomic structure of the large ribosomal subunit at 2.4 A° resolution. *Science* 289 905–920. 10.1126/science.289.5481.905 10937989

[B4] Brimacombe R. Mitchell P. Osswald M. Stade K. Bochkariov D. (1993). Clustering of modified nucleotides at the functional center of bacterial ribosomal RNA. *FASEB J.* 7 161–167. 10.1096/fasebj.7.1.8422963 8422963

[B5] Bujnicki J. M. Rychlewski L. (2002). RNA:(guanine-N2) methyltransferases RsmC/RsmD and their homologs revisited–bioinformatic analysis and prediction of the active site based on the uncharacterized Mj0882 protein structure. *BMC Bioinformatics* 3:10. 10.1186/1471-2105-3-10 11929612 PMC102759

[B6] Burakovsky D. E. Prokhorova I. V. Sergiev P. V. Milón P. Sergeeva O. V. Bogdanov A. A. (2012). Impact of methylations of m2G966/m5C967 in 16S rRNA on bacterial fitness and translation initiation. *Nucleic Acids Res.* 40 7885–7895. 10.1093/nar/gks508 22649054 PMC3439901

[B7] Carrión M. Gómez M. J. Merchante-Schubert R. Dongarrá S. R. Ayala J. A. (1999). mraW, an essential gene at the dcw cluster of *Escherichia coli* codes for a cytoplasmic protein with methyltransferase activity. *Biochimie* 81 879–888. 10.1016/s0300-9084(99)00208-4 10572301

[B8] Clough S. J. Bent A. F. (1998). Floral dip: a simplified method for *Agrobacterium*-mediated transformation of *Arabidopsis thaliana*. *Plant J.* 16 735–743. 10.1046/j.1365-313x.1998.00343.x 10069079

[B9] Fleischmann T. T. Scharff L. B. Alkatib S. Hasdorf S. Schöttler M. A. Bock R. (2011). Nonessential plastid-encoded ribosomal proteins in tobacco: a developmental role for plastid translation and implications for reductive genome evolution. *Plant Cell* 23 3137–3155. 10.1105/tpc.111.088906 21934145 PMC3203423

[B10] Graf M. Arenz S. Huter P. Dönhöfer A. Novácek J. Wilson D. N. (2017). Cryo-EM structure of the spinach chloroplast ribosome reveals the location of plastid-specific ribosomal proteins and extensions. *Nucleic Acids Res.* 45 2887–2896. 10.1093/nar/gkw1272 27986857 PMC5389730

[B11] Green R. Noller H. F. (1999). Reconstitution of functional 50S ribosomes from *in vitro* transcripts of *Bacillus stearothermophilus* 23S rRNA. *Biochemistry* 38 1772–1779. 10.1021/bi982246a 10026257

[B12] Harms J. Schluenzen F. Zarivach R. Bashan A. Gat S. Agmon I. (2001). High resolution structure of the large ribosomal subunit from a mesophilic eubacterium. *Cell* 107 679–688. 10.1016/s0092-8674(01)00546-3 11733066

[B13] Jemiolo D. K. Taurence J. S. Giese S. (1991). Mutations in 16S rRNA in *Escherichia coli* at methyl-modified sites: G966, G967, and G1207. *Nucleic Acids Res.* 19 4259–4265. 10.1093/nar/19.15.4259 1714565 PMC328571

[B14] Keeling P. J. (2013). The number, speed, and impact of plastid endosymbioses in eukaryotic evolution. *Annu. Rev. Plant Biol*. 64 583–607. 10.1146/annurev-arplant-050312-120144 23451781

[B15] Khaitovich P. Tenson T. Kloss P. Mankin A. S. (1999). Reconstitution of functionally active *Thermus aquaticus* large ribosomal subunits with *in vitro* transcribed rRNA. *Biochemistry* 38 1780–1788. 10.1021/bi9822473 10026258

[B16] Kimura S. Suzuki T. (2010). Fine-tuning of the ribosomal decoding center by conserved methyl-modifications in the *Escherichia coli* 16S rRNA. *Nucleic Acids Res.* 38 1341–1352. 10.1093/nar/gkp1073 19965768 PMC2831307

[B17] Krzyzosiak W. Denman R. Nurse K. Hellmann W. Boubik M. Gehrke C. W. (1987). In vitro synthesis of 16S ribosomal RNA containing single base changes and assembly into functional 30S ribosome. *Biochemistry* 26 2353–2364. 10.1021/bi00382a042 3304424

[B18] Lesnyak D. V. Osipiuk J. Skarina T. Sergiev P. Bogdanov A. A. Edwards A. (2007). Methyltransferase that modifies guanine 966 of the 16S rRNA: functional identification and tertiary structure. *J. Biol. Chem.* 282 5880–5887. 10.1074/jbc.M608214200 17189261 PMC2885967

[B19] Li Y. Liu B. Zhang J. Kong F. Zhang L. Meng H. (2019). OHP1, OHP2, and HCF244 form a transient functional complex with the photosystem II reaction center. *Plant Physiol.* 179 195–208. 10.1104/pp.18.01231 30397023 PMC6324237

[B20] Liu X. Rodermel S. R. Yu F. (2010). A *var2* leaf variegation suppressor locus, *SUPPRESSOR OF VARIEGATION3*, encodes a putative chloroplast translation elongation factor that is important for chloroplast development in the cold. *BMC Plant Biol*. 10:287. 10.1186/1471-2229-10-287 21187014 PMC3022910

[B21] Majeran W. Friso G. Asakura Y. Qu X. Huang M. Ponnala L. (2012). Nucleoid-enriched proteomes in developing plastids and chloroplasts from maize leaves: a new conceptual framework for nucleoid functions. *Plant Physiol.* 158 156–189. 10.1104/pp.111.188474 22065420 PMC3252073

[B22] Manduzio S. Kang H. (2021). RNA methylation in chloroplasts or mitochondria in plants. *RNA Biol*. 18 2127–2135. 10.1080/15476286.2021.1909321 33779501 PMC8632092

[B23] Moazed D. Noller H. F. (1990). Binding of tRNA to the ribosomal A and P sites protects two distinct sets of nucleotides in 16S rRNA. *J. Mol. Biol.* 211 135–145. 10.1016/0022-2836(90)90016-F 2405162

[B24] Ngoc L. N. T. Park S. J. Huong T. T. Lee K. H. Kang H. (2021a). N4- methylcytidine rRNA methylation in chloroplasts is crucial for chloroplast function, development, and abscisic acid response in *Arabidopsis*. *J. Integr. Plant Biol.* 63 570–582. 10.1111/jipb.13009 32876986

[B25] Ngoc L. N. T. Park S. J. Cai J. Huong T. T. Lee K. Kang H. (2021b). RsmD, a Chloroplast rRNA m2G Methyltransferase, Plays a Role in Cold Stress Tolerance by Possibly Affecting Chloroplast Translation in *Arabidopsis*. *Plant Cell Physiol.* 62 948–958. 10.1093/pcp/pcab060 34015128

[B26] Porra R. J. (2002). The chequered history of the development and use of simultaneous equations for the accurate determination of chlorophylls a and b. *Photosynth. Res.* 73 149–156. 10.1023/A:1020470224740 16245116

[B27] Pulido P. Zagari N. Manavski N. Gawronski P. Matthes A. Scharff L. B. (2018). CHLOROPLAST RIBOSOME ASSOCIATED supports translation under stress and interacts with the ribosomal 30S Subunit. *Plant Physiol.* 177 1539–1554. 10.1104/pp.18.00602 29914890 PMC6084680

[B28] Rana K. A. Chandr S. Siddiqi M. I. Misra-Bhattacharya S. (2013). Molecular Characterization of an *rsmD*-Like rRNA Methyltransferase from the *Wolbachia* Endosymbiont of *Brugia malayi* and Antifilarial Activity of Specific Inhibitors of the Enzyme. *Antimicrob. Agents Chemother.* 57 3843–3856. 10.1128/AAC.02264-12 23733469 PMC3719755

[B29] Rogalski M. Schöttler M. A. Thiele W. Schulze W. X. Bock R. (2008). Rpl33, a nonessential plastid-encoded ribosomal protein in tobacco, is required under cold stress conditions. *Plant Cell* 20 2221–2237. 10.1105/tpc.108.060392 18757552 PMC2553612

[B30] Schuwirth B. S. Borovinskaya M. A. Hau C. W. Zhang W. Vila-Sanjurjo A. Holton J. M. (2005). Structures of the bacterial ribosome at 3.5 A° resolution. *Science* 310 827–834. 16272117 10.1126/science.1117230

[B31] Sergiev P. Golovina A. Prokhorova I. Sergeeva O. Osterman I. Nesterchuk M. (2011). “Modifications of ribosomal RNA: from enzymes to function,” in *Ribosomes: Structure, Function, and Dynamics*, eds Rodnina M. V. Wintermeyer W. Green R. (Wien, NY: Springer), 97–110. 10.1007/978-3-7091-0215-2_9

[B32] Tokuhisa J. G. Vijayan P. Feldmann K. A. Browse J. A. (1998). Chloroplast development at low temperatures requires a homolog of DIM1, a yeast gene encoding the 18S rRNA demethylase. *Plant Cell* 10 699–711. 10.1105/tpc.10.5.699 9596631 PMC144018

[B33] Urlaub H. Thiede B. Müller E. C. Brimacombe R. Wittmann-Liebold B. (1997). Identification and sequence analysis of contact sites between ribosomal proteins and rRNA in *Escherichia coli* 30 S subunits by a new approach using matrix-assisted laser desorption/ionization-mass spectrometry combined with N-terminal microsequencing. *J. Biol. Chem.* 272 14547–14555. 10.1074/jbc.272.23.14547 9169412

[B34] Wei Y. Zhang H. Gao Z. Q. Wang W. J. Shtykovac E. V. Xu J. H. (2012). Crystal and solution structures of methyltransferase RsmH provide basis for methylation of C1402 in 16S rRNA. *J. Struct. Biol.* 179 29–40. 10.1016/j.jsb.2012.04.011 22561317

[B35] Westrich L. D. Gotsmann V. L. Herkt C. Ries F. Kazek T. Trosch R. (2021). The versatile interactome of chloroplast ribosomes revealed by affinity purification mass spectrometry. *Nucleic Acids Res*. 49 400–415. 10.1093/nar/gkaa1192 33330923 PMC7797057

[B36] Wimberly B. T. Brodersen D. E. Clemons W. M. J. Morgan-Warren R. J. Carter A. P. Vonrhein C. (2000). Structure of the 30 S ribosomal subunit. *Nature* 407 327–339. 11014182 10.1038/35030006

[B37] Xiong H. B. Wang J. Huang C. Rochaix J. D. Lin F. M. Zhang J. X. (2020). mTERF8, a member of the mitochondrial transcription termination factor family, is involved in the transcription termination of chloroplast Gene psbJ. *Plant Physiol*. 182 408–423. 10.1104/pp.19.00906 31685645 PMC6945865

[B38] Yamamoto T. Burke J. Autz G. Jagendorf A. T. (1981). Bound Ribosomes of Pea chloroplast thylakoid membranes: location and release *in vitro* by high salt, Puromycin, and RNase. *Plant Physiol.* 67 940–949. 10.1104/pp.67.5.940 16661797 PMC425805

[B39] Yoo S. D. Cho Y. H. Sheen J. (2007). Arabidopsis mesophyll protoplasts: a versatile cell system for transient gene expression analysis. *Nat. Protoc.* 2 1565–1572. 10.1038/nprot.2007.199 17585298

[B40] Yu Q. B. Lu Y. Ma Q. Zhao T. T. Huang C. Zhao H. F. (2013). TAC7, an essential component of the plastid transcriptionally active chromosome complex, interacts with FLN1, TAC10, TAC12 and TAC14 to regulate chloroplast gene expression in *Arabidopsis thaliana*. *Physiol. Plant.* 148 408–421. 10.1111/j.1399-3054.2012.01718.x 23082802

[B41] Yusupov M. M. Yusupova G. Z. Baucom A. Lieberman K. Earnest T. N. Cate J. H. (2001). Crystal structure of the ribosome at 5.5 A° resolution. *Science* 292 883–896. 11283358 10.1126/science.1060089

[B42] Zhang J. Yuan H. Yang Y. Fish T. Lyi S. M. Thannhauser T. W. (2016). Plastid ribosomal protein S5 is involved in photosynthesis, plant development, and cold stress tolerance in Arabidopsis. *J. Exp. Bot.* 67 2731–2744. 10.1093/jxb/erw106 27006483 PMC4861020

[B43] Zhang L. Pu H. Duan Z. Li Y. Liu B. Zhang Q. (2018). Nucleus-Encoded Protein BFA1 Promotes Efficient Assembly of the Chloroplast ATP Synthase Coupling Factor. *Plant Cell* 30 1770–1788. 10.1105/tpc.18.00075 30012777 PMC6139693

[B44] Zoschke R. Bock R. (2018). Chloroplast translation: structural and functional organization, operational control, and regulation. *Plant Cell* 30 745–770. 10.1105/tpc.18.00016 29610211 PMC5969280

[B45] Zou M. Mu Y. Chai X. Ouyang M. Yu L. J. Zhang L. (2020). The critical function of the plastid rRNA methyltransferase, CMAL, in ribosome biogenesis and plant development. *Nucleic Acids Res.* 48 3195–3210. 10.1093/nar/gkaa129 32095829 PMC7102989

