# Supplementary figures and images for: AtRsmD Is Required for Chloroplast Development and Chloroplast Function in Arabidopsis thaliana
Source: Front Plant Sci. 2022 Apr 25;13:860945. doi: 10.3389/fpls.2022.860945 (PMC9083416; doi:10.3389/fpls.2022.860945)

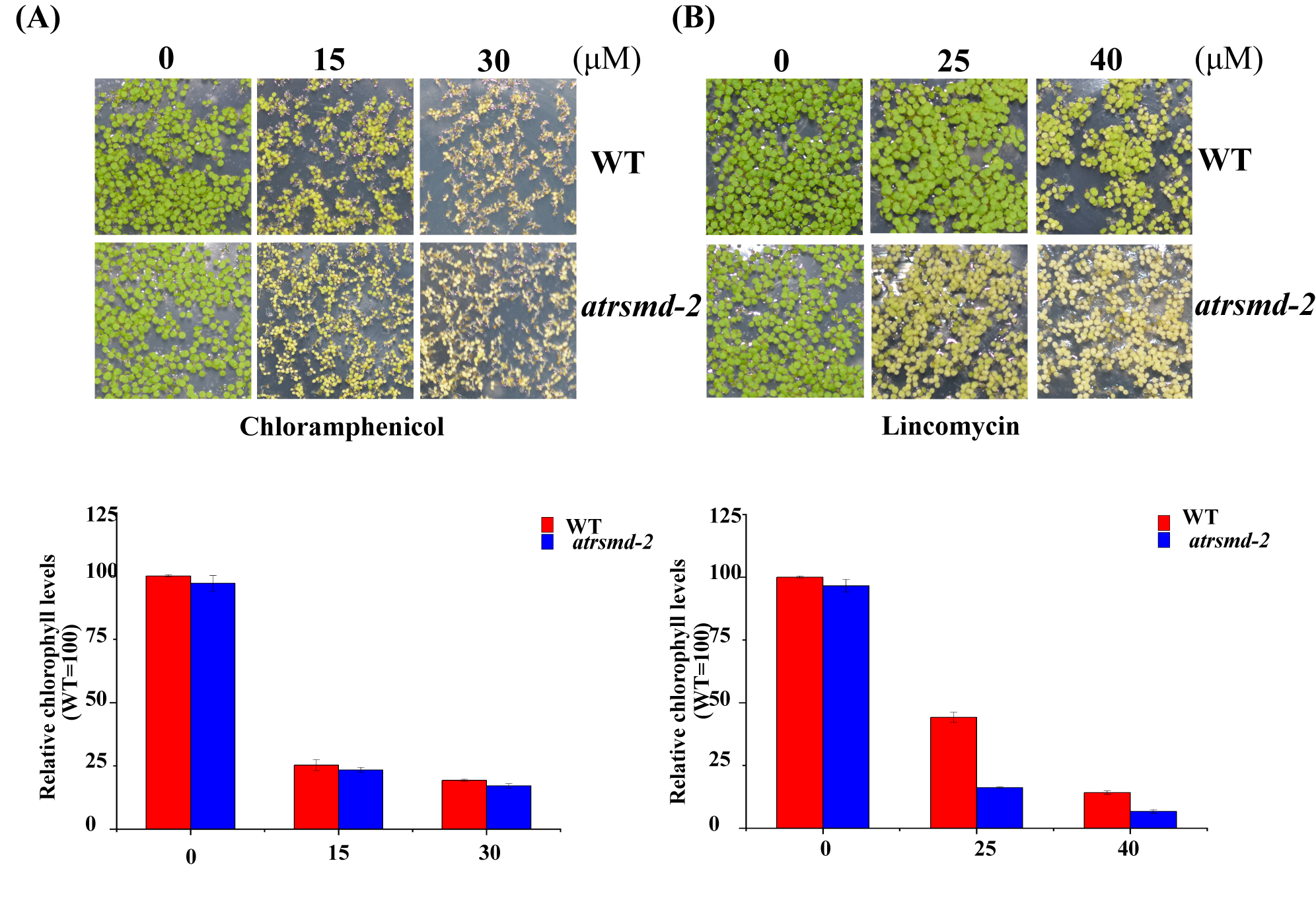

Supplement: Supplementary Figure 1 — Absence of AtRsmD enhances the effects of the translation inhibitors LIN and CAP. (A) Representative images of 5-day-old wild-type (WT) and atrsmd-2 mutant plants germinated on Murashige and Skoog (MS) medium containing the indicated concentrations of LIN. The quantification of the total chlorophyll levels (see section “Materials and Methods”) demonstrates the differences between wild-type and mutant plants grown in the presence of LIN. Relative data are shown (wild-type plants grown in the absence of LIN = 100%), and means ± se values (n = 3) are provided. (B) Representative images of 5-day-old wild-type (WT) and atrsmd-2 mutant plants germinated on Murashige and Skoog (MS) medium containing the indicated concentrations of CAP. The quantification of the total chlorophyll levels (see section “Materials and Methods”) demonstrates the differences between wild-type and mutant plants grown in the presence of CAP. Relative data are shown (wild-type plants grown in the absence of CAP = 100%), and means ± se values (n = 3) are provided. [file Image_1.tif]

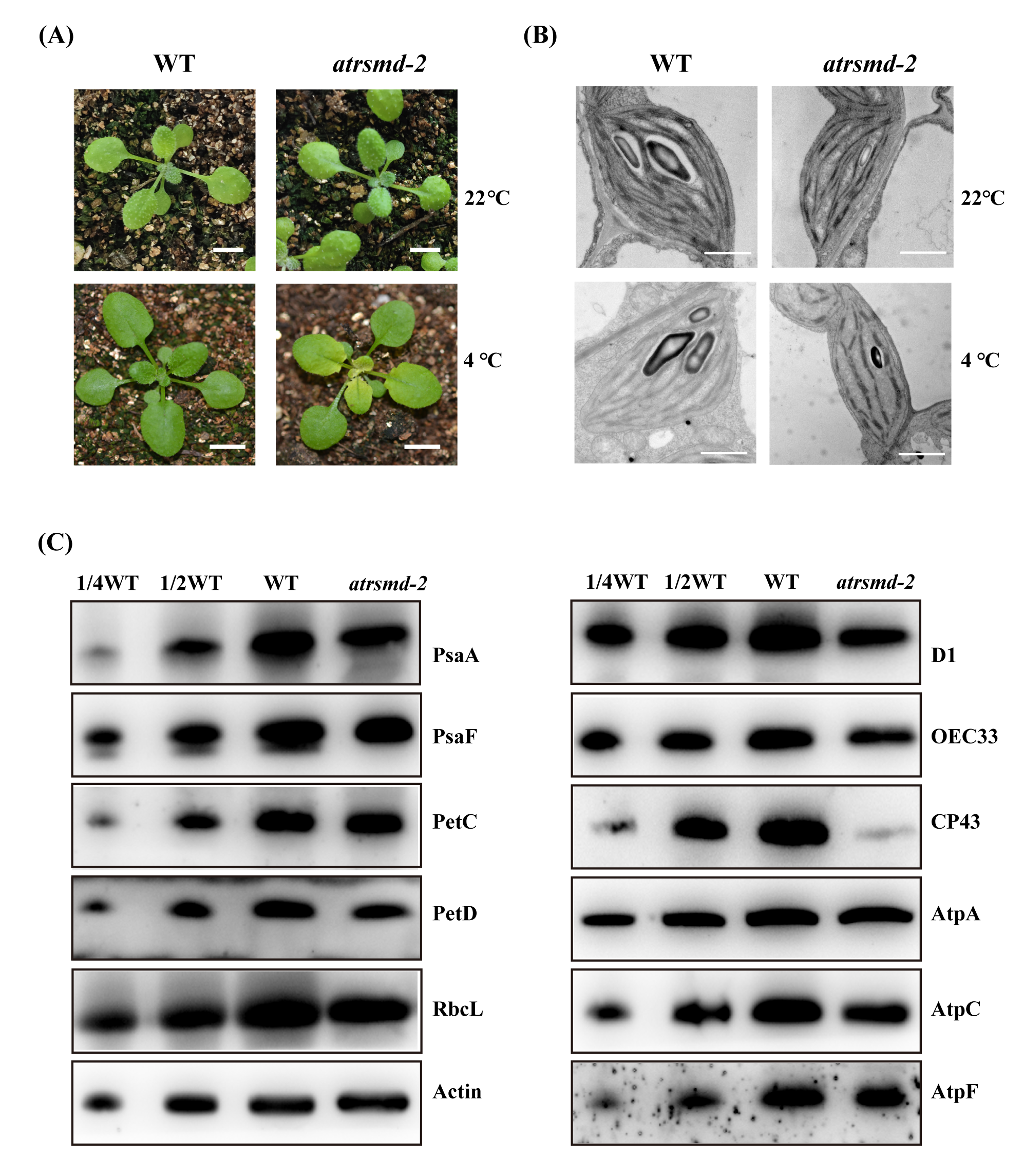

Supplement: Supplementary Figure 2 — Chloroplast development in the wild type and atrsmd-2 mutant treated with cold stress. (A) Phenotype of the wild type and atrsmd-2 mutant treated with cold stress. Bar: 1 cm. (B) Chloroplast ultrastructure observation in the wild type and atrsmd-2 mutant. Bar: 1 μm. (C) Immunoblot analysis of the photosynthetic proteins from the wild type (WT) and atrsmd-2 mutant that were treated with cold stress. Immunoblot analysis of the photosynthetic proteins PsaA, PsaF, D1, CP43, OEC33, AtpA, AtpC, AtpF, PetC, PetD, and RbcL was performed using the corresponding antibodies. Total proteins from the WT samples were loaded at three different concentrations (2.5, 5, and 10 μg), and total proteins from the mutant samples were loaded at 10 μg. [file Image_2.tif]

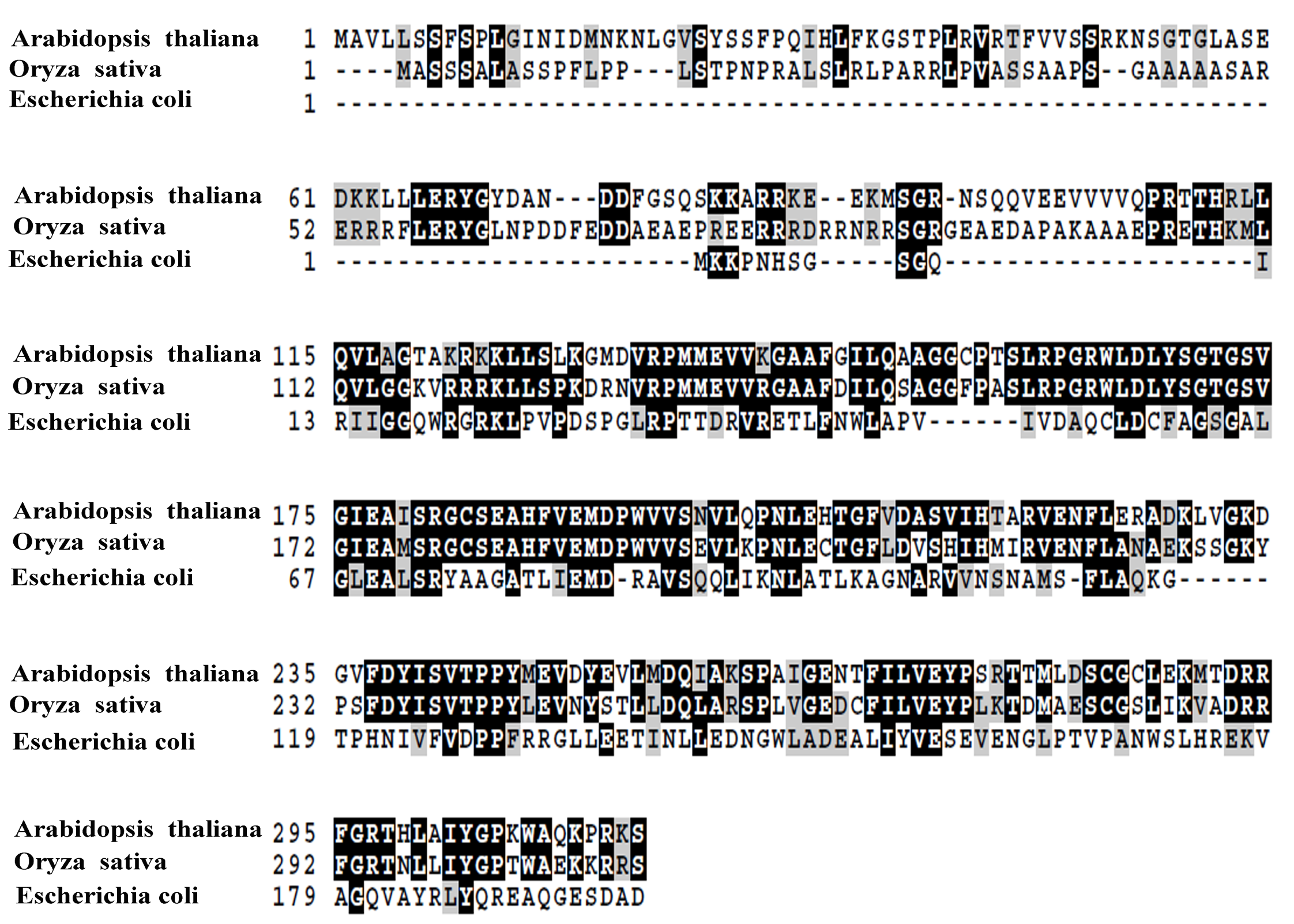

Supplement: Supplementary Figure 3 — Alignment of E. coli RsmD homologous proteins in land plants. Alignment of the amino acid sequence of RsmD homologs in land plants. Sequence identifiers for RsmD homologs are as follows: Arabidopsis thaliana (NP_189487.2), Oryza sativa japonica group (EEE59885.1), and Escherichia coli (EGS2103359.1). [file Image_3.tif]

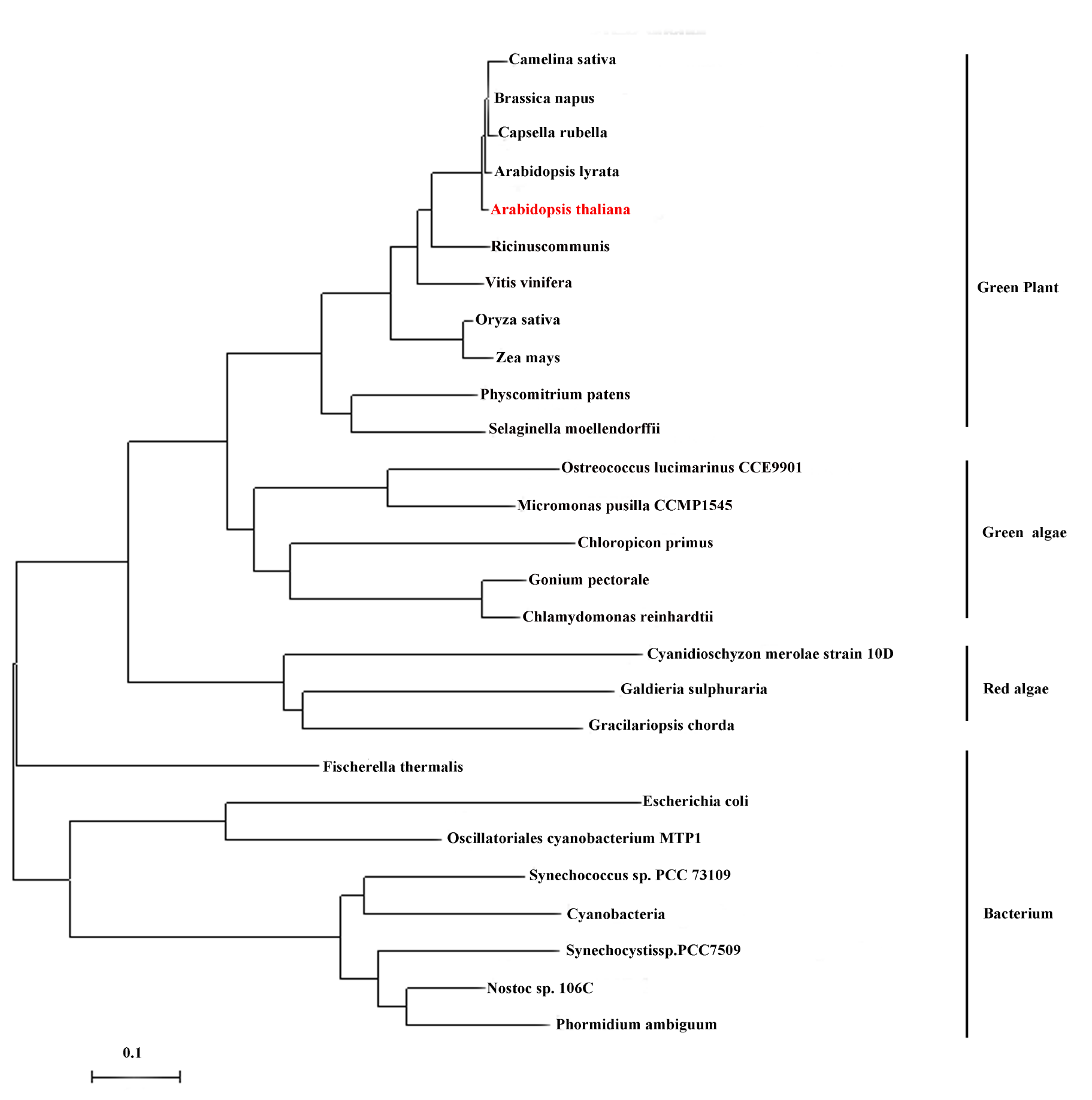

Supplement: Supplementary Figure 4 — Phylogenetic analysis of the AtRsmD protein and its orthologs from different species. The orthologous proteins are listed as follows: Camelina sativa (XP_010514550.1), Brassica napus (XP_013711825.1), Capsella rubella (XP_023639147.1), Arabidopsis lyrata subsp. Lyrata (XP_002877109.1), Arabidopsis thaliana (NP_189487.2), Ricinus communis (XP_015573725.1), Vitis vinifera (XP_002281501.1), Oryza sativa Japonica Group (EEE59885.1), Zea mays (ACG38583.1), Physcomitrella patens (XP_024381071.1), Selaginella moellendorffii (EFJ21750.1), Ostreococcus lucimarinus CCE9901(XP_001417522.1), Micromonas pusilla CCMP1545(XP_003060663.1), Chloropicon primus (QDZ19162.1), Gonium pectoral (KXZ51239.1), Chlamydomonas reinhardtii (XP_042917255.1), Cyanidioschyzon merolae strain 10D (XP_005537355.1), Galdieria sulphuraria (XP_005703506.1), Gracilariopsis chorda (PXF40802.1), Fischerella thermalis (WP_102206177.1), Escherichia coli (EGS2103359.1), Oscillatoriales cyanobacterium MTP1(TAD77760.1), Synechococcus sp. PCC 73109 (WP_062431539.1), Cyanobacteria (WP_044151411.1), Synechocystis sp. PCC 7509 (WP_009631812.1), Nostoc sp. 106C (WP_086756145.1), and Phormidium ambiguum (WP_073593883.1). [file Image_4.tif]

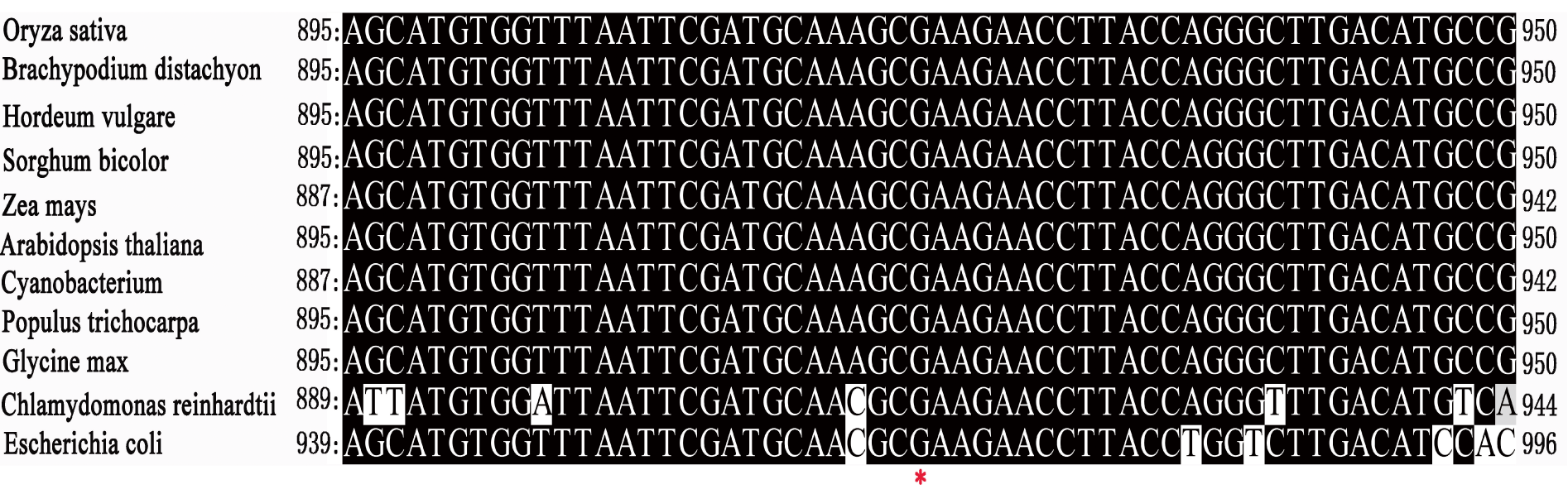

Supplement: Supplementary Figure 5 — Alignment of 16S rRNAs from different organisms. Sequence identifiers for RsmD homologs are as follows: Oryza sativa (MK348618.1), Brachypodium distachyon (LR537486.1), Hordeum vulgare, (MN171392.1), Sorghum bicolor (MK348612.1), Zea mays (MK348606.1), Arabidopsis thaliana (MK353213.1), Cyanobacterium (LN833508.1), Populus trichocarpa (AC208048.1), Glycine max (DQ317523.1), Chlamydomonas reinhardtii (MF083689.2), and Escherichia coli (CP056921.1). [file Image_5.tif]
