# Supplementary material for: AtRsmD Is Required for Chloroplast Development and Chloroplast Function in Arabidopsis thaliana
Source: Front Plant Sci. 2022 Apr 25;13:860945. doi: 10.3389/fpls.2022.860945 (PMC9083416; doi:10.3389/fpls.2022.860945)
Supplement: Supplementary file 6 [file Table_1.DOCX]

| **Supplemental Table S1. Primers used in this study.** | | | | | | | |
| --- | --- | --- | --- | --- | --- | --- | --- |
| **T-DNA insert lines specific primers** | | | | | | | |
| **CS832131** | | | **Lba** | | GCCTTTTCAGAAATGGATAAATAGCCTTGCTTCC | | |
|  |  |  | **LP** | | CTGCAGACGATTTCTCTCTGC | | |
|  |  |  | **RP** | | TAACCGAAGCATCAACAAACC | | |
| **Primers for the genetic complementation experiment** | | | | | | | |
| **At** | | | **Fw** | CGAGATCTGTGGTCTTCCATTGCACGAAAGCTTCC | | | |
|  |  |  | **Rv** | CGGTCGACAGATTTTCTTGGCTTCTGAGCCCATTTTGG | | | |
| **Primers for the Subcellular Localization** | | | | | | | |
| **AtRsmDGFP** | **Fw** | | | | | | CGCATATGATGGCGGTTTTGTTGTCATCTTTC |
|  | **Rv** | | | | | | CGGTCGACAGATTTTCTTGGCTTCTGAGCCCATTTTGG |
| **Primers for Northern blots Analysis** | | | | | | | |
| **4.5S rRNA** | | **Fw** | | | | TCACGGCGAGACGAGCCGTTTAT | |
|  |  | **Rv** | | | | GTTCAAGTCTACCGGTCTGTTAG | |
| **5S rRNA** | | **Fw** | | | | TATTCTGGTGTCCTAGGCGTAGA | |
|  |  | **Rv** | | | | ATCCTGGCGTCGAGCTATTTTTC | |
| **16S rRNA** | | **Fw** | | | | TCTCATGGAGAGTTCGATCCT | |
|  |  | **Rv** | | | | AAAGGAGGTGATCCAGCCGCAC | |
| **23S rRNA** | | **Fw** | | | | GCAAGACCCACCCGTCGAGC | |
|  |  | **Rv** | | | | CGCTCCGCACTTGGCTACCC | |
| ***psbA*** | | **Fw** | | | | ATGACTGCAATTTTAGAGAG | |
|  |  | **Rv** | | | | TTATCCATTTGTAGATGGAGCC | |
| ***rbcL*** | | **Fw** | | | | ATGTCACCACAAACAGAGAC | |
|  |  | **Rv** | | | | CTCCCTGTCTCCTTCAAGTT | |
| **Primers for the AtRsmD gene expression** | | | | | | | |
| **TublinqRT** | | Fw | | | | GATTTCAAAGATTAGGGAAGAGTA | |
|  |  | Rv | | | | GTTCTGAAGCAAATGTCATAGAG | |
| **AtRsmDqRT** | | Fw | | | | ATGGCGGTTTTGTTGTCATC | |
|  |  | Rv | | | | CAGTGGAGTAGAACCCTTAA | |
| **Primers for Chloroplast gene expression** | | | | | | | |
| **PRPS1** | | Fw | | | | ATGGCGTCTTTGGCTCAGCA | |
|  |  | Rv | | | | CGGAGAAACAGAGGCAGATT | |
| **PRPL4** | | Fw | | | | ATGGCTTCTTCTGCAACAGC | |
|  |  | Rv | | | | ACAGAAAGCGGCTTCGAGAC | |
| **PRPL2** | | Fw | | | | CCGAGCACACGCAATGGAGC | |
|  |  | Rv | | | | TTATGACCTCCCCCTCTATG | |
| **psaA** | | Fw | | | | ATGATTATTCGTTCGCCGGA | |
|  |  | Rv | | | | CGAGAGATTTCCTCCAAATC | |
| **psbA** | | Fw | | | | ATGACTGCAATTTTAGAGAG | |
|  | | Rv | | | | GAAACAGGTTCACGAATACC | |
| **psbB** | | Fw | | | | ATGGGTTTGCCTTGGTATCG | |
|  |  | Rv | | | | CGAGTCATGAAAGGTATAACG | |
| **psbC** | | Fw | | | | ATGAAAACCTTATATTCCCT | |
|  |  | Rv | | | | TAAGTTCATTGCTCCGGCCC | |
| **psbD** | | Fw | | | | ATGACTATAGCCCTTGGTAA | |
|  |  | Rv | | | | GGAACTGGCCAATCCATGAG | |
| **atpA** | | Fw | | | | ATGGTAACCATTAGAGCCGA | |
|  |  | Rv | | | | GGATTCTAAATTAAGGGCAA | |
| **atpB** | | Fw | | | | GAGCTCGTATGAGAGTTGGT | |
|  |  | Rv | | | | ACCCAATAAGGCGGATACCT | |
| **atpF** | | Fw | | | | ATGAAAAATTTAACCGATTC | |
|  |  | Rv | | | | TTCTTCTGAATTTCGAATAG | |
| **petA** | | Fw | | | | ATGCAAACTAGAAATACCTT | |
|  |  | Rv | | | | ATATCCACGGGCTTATTAGC | |
| **petD** | | Fw | | | | GAATGATCCTGTATTACGAG | |
|  |  | Rv | | | | GGTAATATTTCCAAAGGAGT | |
| **rbcL** | | Fw | | | | ATGTCACCACAAACAGAGAC | |
|  |  | Rv | | | | CACAGTTGTCCATGTACCAG | |
| **rpoB** | | Fw | | | | ATGCTTGGGGATGAAAAAGA | |
|  |  | Rv | | | | AGCATCTCGTTCTTTTATCA | |
| **16 SrRNA** | | Fw | | | | CGGTATCTGGGGAATAAGCA | |
|  | | Rv | | | | GATTTGACGGCGGACTTAAA | |
| **23S rRNA** | | Fw | | | | CGAGGAAAGGCTTACGGTGG | |
|  | | Rv | | | | AGGTTGTCTCTTGCCTGCC | |
| **5S rRNA** | | Fw | | | | ATTCTGGTGTCCTAGGCGTAG | |
|  | | Rv | | | | ATCCTGGCGTCGAGCTATTTTTC | |
| **4.5S rRNA** | | Fw | | | | GAAGGTCACGGCGAGACGAG | |
|  | | Rv | | | | GTTCAAGTCTACCGGTCTGTTAG | |
